# Structure determination by cryoEM at 100 keV

**DOI:** 10.1073/pnas.2312905120

**Published:** 2023-11-27

**Authors:** Greg McMullan, Katerina Naydenova, Daniel Mihaylov, Keitaro Yamashita, Mathew J. Peet, Hugh Wilson, Joshua L. Dickerson, Shaoxia Chen, Giuseppe Cannone, Yang Lee, Katherine A. Hutchings, Olivia Gittins, Mohamed A. Sobhy, Torquil Wells, Mohamed M. El-Gomati, Jason Dalby, Matthias Meffert, Clemens Schulze-Briese, Richard Henderson, Christopher J. Russo

**Affiliations:** ^a^Medical Research Council (MRC) Laboratory of Molecular Biology, Cambridge CB2 0QH, United Kingdom; ^b^Newcastle University, Newcastle upon Tyne NE2 4HH, United Kingdom; ^c^King Abdullah University of Science and Technology, Thuwal 23955, Saudi Arabia; ^d^York Probe Sources Ltd., York YO26 6QU, United Kingdom; ^e^JEOL U.K. Ltd., Welwyn Garden City AL7 1LT, United Kingdom; ^f^DECTRIS Ltd., 5405 Baden-Daettwil, Switzerland

**Keywords:** cryoEM, structure determination, 100 keV, TEM, electron microscopy

## Abstract

Electron cryomicroscopy can, in principle, determine the structures of most biological molecules but is currently limited by access, specimen preparation difficulties, and cost. We describe a purpose-built instrument operating at 100 keV—including advances in electron optics, detection, and processing—that makes structure determination fast and simple at a fraction of current costs. The instrument attains its theoretical performance limits, allowing atomic resolution imaging of gold test specimens and biological molecular structure determination in hours. We demonstrate its capabilities by determining the structures of eleven different specimens, ranging in size from 140 kDa to 2 MDa, using a fraction of the data normally required. CryoEM with a microscope designed specifically for high-efficiency, on-the-spot imaging of biological molecules will expand structural biology to a wide range of previously intractable problems.

## Significance

A purpose-built electron cryomicroscope allows rapid, high-resolution, and uncostly structure determination. We describe several advances including a 100-keV field emission gun, a low aberration objective lens with cryobox, and high-speed, high-efficiency electron detector all designed for efficient structure determination by single-particle cryoEM. Together they mean that an electron cryomicroscope can be constructed that is an order of magnitude cheaper than current models, and in practice, it reduces the time and effort required for structure determination, even compared with our own state-of-the-art 300 keV microscopes. This will help set the path for the future development of experimental structural biology and help drive the continuing exponential growth in the number of structures determined by cryoEM.

Determining the accurate atomic structure of molecules underpins many of the current advances in biology. The methods for determining structures by electron cryomicroscopy (cryoEM) have accelerated in recent years (1), with advances in microscope hardware being the key to sustained improvements in the field (2). Furthermore, recent advances in structure prediction methods have accelerated, not replaced, experimental methods for determining structures (3). Yet most of the hardware in microscopes used for structure determination was designed for other applications in physics and materials science and comes at high cost. This has limited the widespread adoption of cryomicroscopy by most biology labs since the instrumentation remains prohibitively expensive and resources have thus far been concentrated in the few centres and large user facilities able to afford them (4). Part of the reason for this is that the evolution of cryoEM has involved solving a succession of technical problems, each of which was rate-limiting to progress in the field (5, 6). Problems including stage stability, poor vacuum near the specimen causing ice contamination, electron optical aberrations, low source brightness, coherence and stability, and especially poor electron detector efficiency have meant that the field unconsciously evolved to favour higher and higher electron energies, with their associated high costs. Solving all these problems has edged up the complexity of current installations incrementally to reach the current situation where a single state-of-the-art cryoEM instrument costs many millions of dollars. Recent work showed that there is an optimum energy to use for imaging most biological molecules and structures, and surprisingly, it is three times lower than the most popular current energy (300 keV) (7). At this stage, it was reasonable to ask which of the innovations in electron microscopy are essential to achieve good quality cryoEM structure determinations, and is it possible to do as well as current instruments but at much lower complexity and cost?

## Results

**Fig. 1.**
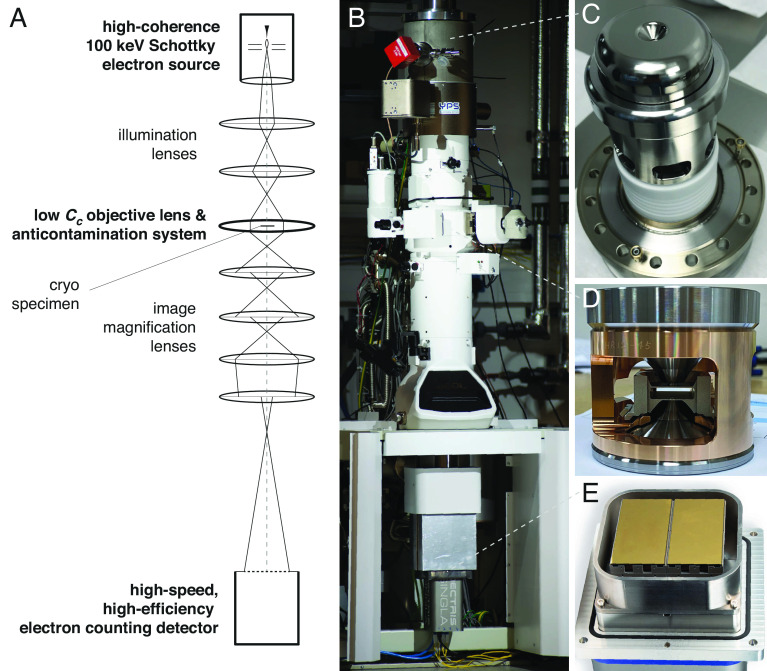
The design of a purpose-built 100-keV electron cryomicroscope for structure determination. A diagram of the microscope components (*A*) highlights the key elements identified for improvement in this work. Images of the complete microscope (*B*) with inset views of the new Schottky field emission gun (*C*), low $C_{c}$ objective lens and liquid nitrogen anticontamination system (*D*), and the high-speed, high-efficiency direct electron detector (*E*). The column is 2.7 m tall; together with the supporting components and a specimen preparation bench, it occupies a room that is 3 × 4 × 3.2 m.

In this work, we have implemented the key elements of an electron cryomicroscope that are required for molecular structure determination with an order of magnitude reduction in the cost versus current state-of-the-art microscopes (Fig. 1). Given the increase in signal per unit radiation damage on most biological specimens demonstrated in ref. 7, the first critical improvement to the cryomicroscope is an electron source designed specifically for 100-keV energy. We have developed a Schottky field emission gun (FEG) (8) as an electron source (Fig. 1*C*), the details of which were described recently (9). The key properties of the source for imaging molecules are a stable emission of electrons, with high brightness / spatial coherence as well as low energy spread / high temporal coherence. The source has a compact power supply that is stable to less than 100 mV at 100 kV and does not need sulphur hexafluoride (a highly regulated, potent greenhouse gas) which is normally necessary to prevent arcing in higher energy sources. This reduces a major component of the cost, complexity, and environmental impact of maintaining the instrument over time.

**Fig. 2.**
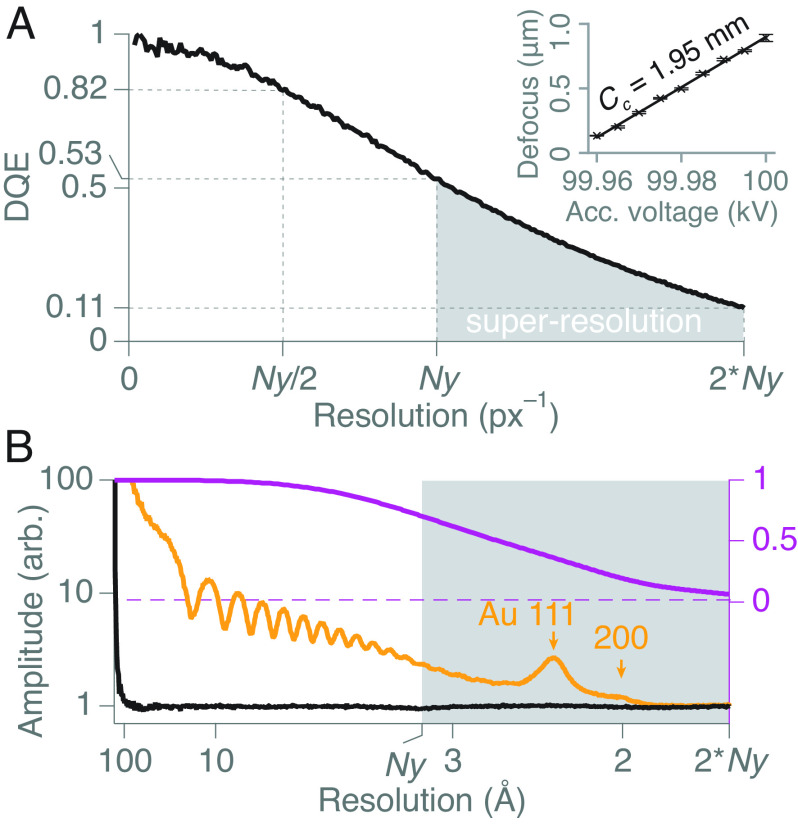
Imaging performance of the 100-keV microscope. The measured DQE as a function of spatial frequency for the detector and real-time electron counting algorithm is shown in *A*. Ny indicates the physical Nyquist frequency (∼1/[2 pixels] for this detector), and the region beyond Ny is taken as “super-resolution.” A measurement of the chromatic aberration ($C_{c}$) of the imaging system is inset. The azimuthal average of Fig. 3*D* is shown in *B* (gold), with the average from a uniform electron flux image under the same conditions (black). Peaks at 2.3 and 2.0 Å are indicated, showing the resolution is consistent with that expected from the temporal coherence envelope set by the source energy spread and the chromatic aberration of the imaging lenses (plotted in purple).

The second critical choice was an objective lens with a low chromatic aberration coefficient, $C_{c}$, that still leaves space for a suitable cryoanticontamination device to prevent residual water in the column from building up on the cold specimens as ice (Fig. 1*D*). The $C_{c}$ is the key parameter of the lens design since other spatial aberrations are effectively corrected in software during processing of the data (10, 11) for the resolutions of interest, thus eliminating the need for expensive and complicated hardware correctors for this purpose. In addition, algorithms for Ewald sphere correction of the data after collection also eliminate the increase in Ewald sphere curvature as a concern at lower energies (12–14). To achieve these constraints, an existing high-resolution (HR) pole-piece originally designed for materials science applications was modified by JEOL to include a cryogenic anticontaminator reaching stable temperatures near 80K surrounding the specimen, albeit with a limited range of specimen tilt angles (±15 ${}^{{}^{\circ}}$). Together, this provided a measured $C_{c}$ of 1.9 mm (including the effects of the projection lenses) with a water ice contamination rate of under 7 Å per hour. This results in a chromatic envelope amplitude function (15, 16) with a value greater than 75% at 3 Å resolution (purple curve in Fig. 2*B*) and a practical working time for imaging frozen specimens of more than 12 h.

The third essential feature was a high detective quantum efficiency (DQE) detector (Fig. 1*E*). When work on this microscope began, such a detector was not available. In previous efforts to show the potential for cryoEM at 100 keV (17), we had to use a repurposed hybrid-pixel X-ray detector (DECTRIS EIGER X) with only 0.5 megapixels. In this work, we have used a detector that is twice the size, (DECTRIS SINGLA with 1.0 megapixels) developed especially for cryoEM at 100 keV. To maximise the detection efficiency and speed of the detector, we implemented a hardware and software system capable of detecting the arrival time and location of each electron within each pixel in real time, with a time resolution of 0.22 ms. This allowed exposures with a flux of about 150 pixels per electron per 0.22 ms frame, and a DQE of 98% at zero spatial frequency, 53% at the physical Nyquist frequency and 11% at twice Nyquist (Fig. 2*A*). This was in agreement with theoretical expectations for the sensitivity of the pixels and, to date, is the highest efficiency and speed reported for any large area imaging detector designed for cryoEM at any energy (18–21). The imaging system includes an in-house developed graphical user interface for microscope operation and data collection and was integrated with control over the microscope optics needed to acquire, evaluate, and record low-dose images in real time with ease. The technical details of this system will be reported separately.

**Fig. 3.**
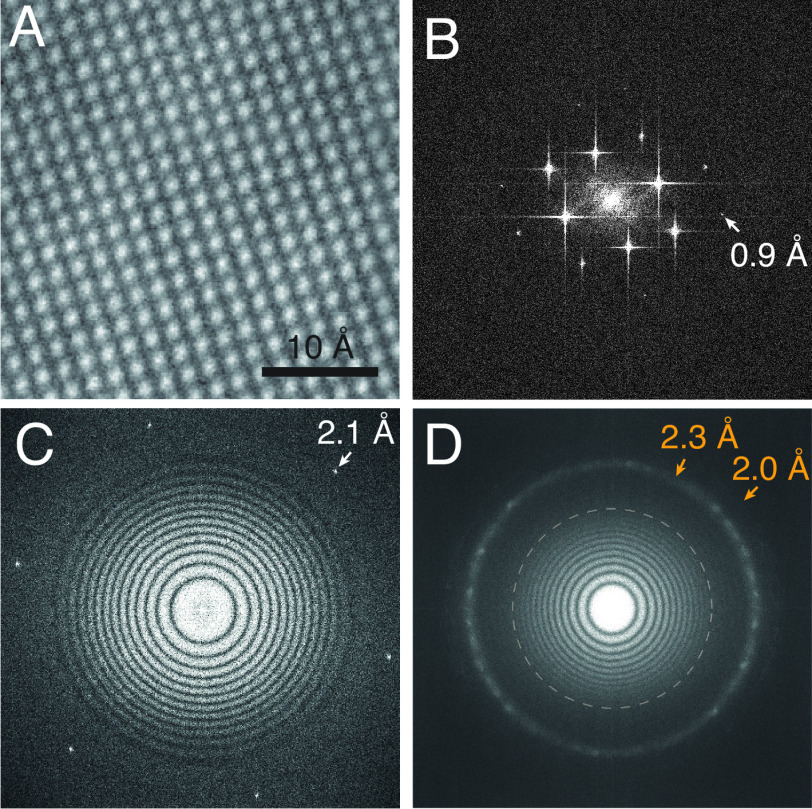
Atomic resolution optics in a simple 100-keV microscope. A region of a high magnification micrograph of a thin gold foil imaged perpendicular to the ⟨200⟩ axis is shown in *A* (complete micrograph in *SI Appendix*, Fig. S1). The amplitude of a 2D FFT of the complete micrograph is shown in *B* where the maximum spatial frequency in each direction is 1/0.5 Å${}^{-1}$. All of the lattice reflections to 1 Å resolution are visible, with the ⟨420⟩ at 0.9 Å just visible and indicated by an arrow. The amplitude of a 2D FFT of single-layer graphene coated with a thin layer of amorphous carbon taken with a 0.8 Å magnified pixel size is shown in *C*. The ⟨100⟩ set of hexagonal reflections from the graphene lattice at 2.1 Å are resolved in all directions with the phase-contrast Thon rings extending to this limit. The amplitude of a 2D FFT of gold on amorphous carbon *D*, imaged with a 1.6 Å magnified physical pixel size shows rings for the ⟨111⟩ and ⟨200⟩ reflections at 2.3 and 2.0 Å at resolutions beyond the physical Nyquist limit.

To demonstrate the optical performance of the microscope, images of three radiation-insensitive, nonbiological specimens were analysed (Fig. 3). An image and Fourier transform of an oriented gold crystal showing the face-centred cubic (fcc) lattice projection with the ⟨200⟩ spacing at 2.04 Å and higher orders out to 0.9 Å are shown in Fig. 3 *A* and *B* and *SI Appendix*, Fig. S1. A graphene monolayer image transform showing its 2.1 Å spacing is shown in Fig. 3*C*, and the image transform of a super-resolution image (using the same pixel size used below for biological cryoEM) of a gold/palladium-shadowed cross-grating is shown in Fig. 3*D*. The image shown in Fig. 3*D* is analysed more quantitatively in Fig. 2*B* where the gold ⟨111⟩ (2.35 Å) and ⟨200⟩ (2.04 Å) super-resolution peaks in the radial amplitude spectrum are clearly visible alongside the theoretical amplitude chromatic envelope function calculated from the known $C_{c}$, electron energy, and energy spread for the instrument (*SI Appendix*). Taken together, these images demonstrate that the electron optical performance of the microscope, in use, reaches the physical limits set by the source coherence, detector efficiency, and lens aberrations.

**Fig. 4.**
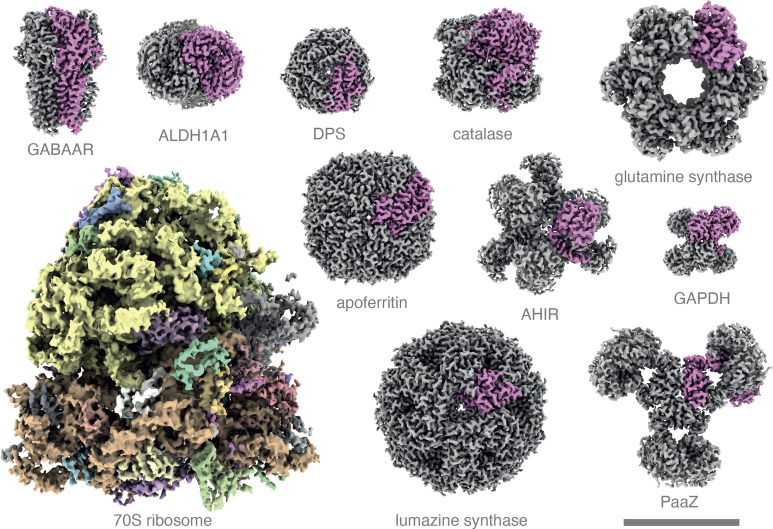
Eleven structures determined at 100 keV. The reconstructed 3D scattering potential for each macromolecular complex is rendered as a surface at a representative contour level, with individual subunits rendered in distinct colours. Details of structure determination including the full names of each complex can be found in *SI Appendix*. Scale bar is 100 Å and applies to all the structures.

To investigate the performance of the microscope in practical biological structure determination, we chose eleven macromolecular specimens with different sizes and symmetries to show that the capabilities of the instrument span the full range of molecular structures for which the single-particle cryoEM method is ideally suited. We determined eleven structures (Fig. 4 and *SI Appendix*, Table S1 and Figs. S2–S13), with three having four identical molecular subunits, three having twelve identical subunits, and one structure each having 1, 5, 6, 24, and 60 subunits. The resolutions were all sufficient to build accurate atomic models (2.6–4.5 Å), and a majority of the structures (7 of 11) extended to beyond physical Nyquist resolution, indicating the power and importance of combined detector and optical performance. Two of the others were essentially at Nyquist resolution (catalase and glutamine synthetase at 3.4 Å), with only PaaZ and the 70S ribosome at 0.9 Nyquist. Resolution was estimated in two ways, first by the usual Fourier shell correlation (FSC) between the two half-maps at 0.143 (22) and second from the map-model FSC at a correlation coefficient at 0.5 (23). The fact that these two independent resolution estimates agree precisely further validates the quality of the structure determinations. When compared to the range of atomic structures determined by cryoEM in the PDB in recent years, we can therefore conclude that the system overall works well for nearly all specimens amenable to single-particle cryoEM. It is notable that all the structures were obtained with a single day of data collection where the number of one megapixel images in each case was between 350 and 500, equivalent to 20-30 images from the current generation of 16-megapixel direct electron detectors in use at 300 keV. Full details of each structure are given in supplementary information (*SI Appendix*, Figs. S2–S12), alongside illustrations of map-model superpositions showing interesting features in each structure (*SI Appendix*, Fig. S13). These include the haem group in catalase and the anaesthetic drug etomidate bound to the membrane-bound GABA${}_{A}$ receptor.

## Discussion

The collection of several hundred images for each specimen in this work involved manual data collection over a few hours at a rate of up to 60 images per hour, which is a data throughput of about 150× less than automated microscopes with 16-megapixel detectors. But this was not as disadvantageous as it might first seem. For example, more automated computer control systems often collect significant numbers of low-quality images from regions of the specimen with amorphous ice that is too thick or too thin, or have other problems. In contrast, these regions are easily skipped during a manual data collection session where the user can see the quality of every image, resulting in a higher success rate for good images. The ability to rapidly load, assess, and unload (ready for another specimen in about 15 min using the current system) means that it is convenient for screening specimens with immediate feedback. It allows multiple rounds of *plunge, image,* and *evaluate* to be conducted in a single morning, thus directly addressing the most limiting factor in modern cryoEM—the quality of the specimen. It also allows immediate collection of datasets suitable for high-resolution structure determination in a few hours as soon as a good specimen is made, thus closing the loop between specimen preparation, optimisation, and data collection to hours rather than days or weeks. This is a key advantage for structure determination in practice that is lost when grids need to be stored and transported to a facility to be imaged long after they are made.

Alongside the work we report above, we also collected equivalent numbers of images on a ThermoFisher Krios with Falcon 4 or 4i detectors at 300-keV electron energy, but using 0.4 or 0.5 Å pixel sizes instead of the 1.65-Å magnified pixel size we used at 100 keV. Each image covered roughly the same specimen area and had equivalent numbers of particles. The resolution obtained was consistently slightly better (10–25%) using the Krios data, meaning that the optical performance of this prototype has not yet surpassed that of the current state-of-the-art cryoEM at 300 keV. There are two likely reasons for this. First, the use of smaller pixel sizes on the Krios at 300 keV with much bigger detectors means that the DQE at 3 Å resolution is around 85%, whereas it is only about 50% under the bigger pixel size/electron-optical conditions we used in the present work. In addition, the increased number of pixels in 300 keV increases the contrast transfer function (CTF) estimation accuracy via the DQE. This is entirely down to the number of pixels available in the detector, and once larger area detectors with equivalent efficiencies are available at 100 keV, this advantage will be removed (*SI Appendix*, Fig. S14*A*). Second, while the energy spread, ΔE, on our 100-keV FEG at 0.7 eV is approximately the same as on a Krios; the 3× lower primary electron energy E means that the ratio ΔE/E is 3× higher, which causes the chromatic envelope function to reduce contrast in spite of our choice of an objective lens with a $C_{c}$ of only 1.9 mm (*SI Appendix*, Fig. S14*A*). These two present advantages on 300-keV microscopes mean that the signal-to-noise ratio at 2.5- to 3.5-Å resolution in our images is still slightly lower than that on high-end, state-of-the-art electron cryomicroscopes. Further effort to reduce the $C_{c}$ at 100 keV with a purpose-built objective lens and specimen loading system for cryomicroscopy would offer a cost-effective way to improve temporal coherence, but other technologies might also be useful (*SI Appendix*, Fig. S14*B*). A low $C_{c}$ objective lens combined with detectors with more pixels and equivalent performance to that demonstrated here would give optical performance equivalent to or surpassing current state-of-the-art at 300 keV, and with the distinct advantage of more signal per unit radiation damage at all resolutions (7). Finally but of major practical importance, once the hardware and optics are optimised, data acquisition could be automated using existing hardware and methods similar to those in place now on high-end microscopes (24, 25) to allow >600 instead of ∼60 images per hour. This combined with a 4× or larger detector would then give datasets suitable for atomic structure determination in a few minutes once a good specimen is made.

We have shown how accurate 3D molecular structures can be obtained conveniently and cost effectively by cryoEM at 100 keV. We anticipate that many of the advances in electron imaging technology described here will also benefit the materials science and low-temperature physics communities, particularly as the need to image a wide array of radiation-sensitive specimens like materials in lithium battery electrodes and devices designed for quantum computation increases (26). We hope that with only two further improvements described here, the efficiency of determination of high-resolution molecular structures with a relatively inexpensive 100-keV electron cryomicroscope will be superior to anything available now, and automation of the data collection on optimised hardware will soon follow. Looking forward, it is becoming clear that cryoEM in structural biology might evolve in two directions, namely lower energy, higher resolution microscopes for single-particle analysis alongside higher-energy instruments for tomography and in situ imaging, with the possibility of economy and luxury model instruments to cater for all needs.

## Materials and Methods

### Microscope Characterization.

#### Chromatic aberration measurement.

The coefficient of chromatic aberration ($C_{c}$) for the microscope used in this work was measured using the dependence of the defocus on the electron accelerating voltage. For this measurement, a standard cross-grating grid (Agar AGS106L) was used (27). The specimen was brought to around 1 μm below the eucentric height, and then micrographs were acquired at 500,000× nominal magnification as the accelerating voltage was ramped down from 100,000 V to 99,960 V and back up to 100,000 V in steps of 5 V. Three micrographs were acquired at each accelerating voltage. The pixel size at this magnification was calibrated to be 0.848 ± 0.004 Å using a HexAuFoil grid (28) and the magCalEM program (29). The micrographs were motion corrected using MotionCorr2 (30) and their CTFs were estimated using CTFFIND-4.1 (10). The $C_{c}$ was determined from the dependence of the defocus z on the accelerating voltage V:

[1] $$\begin{array}{c} \Delta z=C_{c}\frac{\Delta V}{V_{0}}, \end{array}$$

where Δz is the change in defocus caused by a change in the accelerating voltage of ΔV around an initial value of $V_{0}\;=\;$100,000 V.

#### Imaging of graphene and gold test specimens.

Oriented gold (Agar AGS135) was imaged in a room temperature holder at nominal magnification of 1.5 M× corresponding to a magnified pixel size of 0.27 Å. A specimen of monolayer graphene on an UltrAuFoil 0.6/1.0 specimen support was imaged in a room temperature holder at 500 k× magnification corresponding to a magnified pixel size of 0.8 Å. A standard cross grating (Agar AGS106L) was imaged at 250 k× magnification with super-resolution using the same settings as for all cryoEM datasets (other than the 70S which was at lower magnification).

#### Image processing.

The Fourier transform amplitudes of the images of graphene (*C*) and gold (*D*) shown in Fig. 3 were obtained by selecting 1,024 or 2,048 square areas from the centre of each normal or super-resolution SINGLA image, respectively, then treated with the stand-alone MRC image processing program taperedge to remove discontinuities at the edges, and finally noise-whitened by dividing by the radial amplitude of a similar image with no specimen, before being Fourier transformed and displayed using Ximdisp (31). The 1D plots of the gold super-resolution image and its blank control (Fig. 2*C*) show the output of the program raddencalc, which was used to calculate the average radial amplitude of the image in Fig. 3*D*.

#### Coherence envelope calculations.

For a ΔE of −1.8 to +1.8 eV in 0.05 eV steps, the change in focus Δf is calculated as:

[2] $$\begin{array}{c} \Delta f=C_{c}\left(\frac{\Delta E}{E}\right), \end{array}$$

where E is the primary beam energy. The CTF is calculated for each Δf using the following:

[3] $$\begin{array}{c} CTF=\sqrt{1-W^{2}}\sin\chi -W\cos\chi , \end{array}$$

where W is the amplitude contrast, measured to be 0.07 for 100 keV (32), and χ is:

[4] $$\begin{array}{c} \chi =\frac{\pi }{2}\left(C_{s}\lambda^{3}q^{4}+\left(2\left(\Delta z+\Delta f\right)\lambda q^{2}\right)\right), \end{array}$$

with $C_{s}$ the spherical aberration coefficient, λ the wavelength, q the spatial frequency, and Δz the nominal defocus, set to −2.5 μm.

The experimentally measured energy spread of the electron source is then used to calculate a weighted average CTF. The position of the peaks and troughs in the average CTF are identified and the amplitude of these are plotted against spatial frequency and taken as the temporal coherence envelope function, T(f), which is plotted in Fig. 2*B* and *SI Appendix*, Fig. S14 for the microscope described.

### Preparation of Biological Specimens.

#### DPS.

The K27A mutation in DPS (DNA protection from starvation enzyme) was introduced into wild-type *Escherichia coli* DPS(2-167) by QuickChange mutagenesis. The pET15b construct harbours an N-terminal hexahistidine tag and TEV proteolytic site (Ser2 in the P1’ site). The expression vector was transformed into BL21-CodonPlus(DE3)-RIL (Stratagene) and selected on ampicillin-containing LB-agar. 2×YT${}_{\;}\text{Amp}$ medium was inoculated with a single colony and cultured overnight at 37 ${}^{{}^{\circ}}$C with shaking. TB${}_{\;}\text{Amp}$ medium was inoculated 1:50 (v/v) with preculture and grown at 25 ${}^{{}^{\circ}}$C. At OD${}_{\text{600}}\;\text{nm}=0.8$, DPS(K27A) expression was induced with 0.1 mM IPTG for 18 h. Cells (∼20 g/L) were harvested by centrifugation and resuspended 1:1 (w/v) in lysis buffer (20 mM HEPES/Na${}^{+}$ pH 7.5 at 4 ${}^{{}^{\circ}}$C, 500 mM NaCl, 5 mM MgCl${}_{2}$). Cell suspensions were either processed immediately or flash frozen in liquid nitrogen and stored at −80 ${}^{{}^{\circ}}$C. Cells from 1 L of culture were diluted 1:4 (w/v) with lysis buffer and supplemented with 5 mM MgCl${}_{2}$, 20 μg/mL DNase I, 100 μg/mL lysozyme, and protease inhibitor cocktail tablets (Roche) and allowed to stir on ice for 30 min. Cells were lysed by sonication for 5 min at a 33% duty cycle (3 s on; 6 s off) and clarified by centrifugation at 50,000×*g* for 20 min. The supernatant was passed through a 0.45 μm filter and supplemented with 10 mM imidazole before loading onto a 5 mL HisTrap FF column (GE Healthcare) at 2 mL/min at 4 ${}^{{}^{\circ}}$C. The column was washed with 20 column volumes (CV) of lysis buffer supplemented with 40 mM imidazole before eluting with 5 CV of buffer containing 500 mM imidazole. The eluate was treated with 5 mg of TEV protease (+0.5 mM TCEP) and transferred to 3.5K MWCO Snakeskin dialysis tubing (Thermo Fisher Scientific). Imidazole was removed by dialysis against 20 mM HEPES/Na${}^{+}$ pH 7.25 at 20 ${}^{{}^{\circ}}$C, 100 mM NaCl, 5 mM MgCl${}_{2}$, and 0.5 mM TCEP with two changes of buffer. In order to remove any residual DNA-bound DPS, the sample was passed through a 5-mL HiTrap DEAE FF column (GE Healthcare). The flowthrough was supplemented with 10 mM imidazole and subjected to negative chromatography on a 5-mL HisTrap FF column. The final flowthrough was concentrated to ∼1.5 mL using a 10K MWCO Amicon Ultra-15 concentrator (MilliporeSigma). The sample was applied at 0.5 mL/min onto a Superdex 200 Increase 10/300 GL column (GE Healthcare) equilibrated in 10 mM HEPES/Na${}^{+}$ pH 7.5 at 4 ${}^{{}^{\circ}}$C, 100 mM NaCl, sans MgCl${}_{2}$. DPS eluted predominantly as a single major peak with a calibrated retention volume equivalent to 211 kDa or 5.1-nm Stokes radius. Of note, 3 μL of the specimen at 1.5 mg/mL was applied to a 0.6/1.0 UltrAuFoil specimen support, previously exposed to an Ar:O plasma (9:1) for 90 s using a Fischione 1070 plasma chamber. The grid was blotted for 10 s with filter paper and was then immediately plunge-frozen into liquid ethane, kept at 93 K in a temperature-controlled cryostat (33), using a manual plunger in a 4 ${}^{{}^{\circ}}$C cold room.

#### 70S ribosome.

*E. coli* 70S ribosomes were isolated by ultracentrifugation over a sucrose cushion. Briefly, a preculture of *E. coli* strain BL21 (DE3) pLysS was grown overnight in 5 mL LB supplemented with 0.5% glucose at 37 ${}^{{}^{\circ}}$C with shaking at 200 rpm. A 0.5 L volume of LB, supplemented with 0.5% glucose and chloramphenicol, was inoculated with this preculture, and the cells were grown at 37 ${}^{{}^{\circ}}$C with shaking at 200 rpm until optical density at 600 nm of 0.2. The cells were then harvested by centrifugation at 5,000 rpm for 20 min at 4 ${}^{{}^{\circ}}$C. The cell pellet was resuspended in 20 mL of ice-cold physiological salt buffer (50 mM HEPES pH 7.5, 100 mM potassium acetate, and 50 mM magnesium acetate) and centrifuged again at 3,900 rpm for 10 min at 4 ${}^{{}^{\circ}}$C. The cell pellet (1.3 g) was resuspended in 1.8 mL of lysis buffer (physiological salt buffer + 0.5% TritonX-100 + 2 mM CaCl${}_{2}$ + 2 mM MgCl${}_{2}$), and 200 μL of lysozyme from 10 mg/mL stock were added to disrupt the bacterial cell wall. The suspension was supplemented with 1 cOmplete mini EDTA-free protease inhibitor tablet (Sigma-Aldrich) and incubated for 10 min at room temperature, after which 100 μL of DNase I from 2 mg/mL stock were added, and the incubation continued for another 5 min. After one cycle of freezing in liquid nitrogen and thawing in a room-temperature water bath, the cell debris was pelleted from the suspension by centrifugation at 14,000 rpm for 30 min at 4 ${}^{{}^{\circ}}$C. The supernatant, a transparent liquid with a yellow tint, was collected, and 1 mL was carefully layered over a 2 mL 20% (w/v) sucrose cushion (in physiological salt buffer). This was ultra-centrifuged at 100,000 rpm for 30 min at 4 ${}^{{}^{\circ}}$C. The pellet was resuspended in 100 μL of ice-cold physiological salt buffer. The concentration, measured by absorbance at 260 nm, was 46 mg/mL, and the $A_{260}/A_{280}$ ratio was 1.8, as expected for ribosomes. The specimen was diluted 10-fold (to 4.6 mg/mL) for cryoEM grid preparation. An UltrAuFoil R 0.6/1 grid, coated with monolayer graphene, produced in-house as previously described (34) was used to vitrify the specimen. The surface was rendered hydrophilic by exposure to 3 min of hydrogen plasma. Two 3 μL droplets of the specimen were applied to the graphene side of the grid and blotted for 8 s. The grid was then immediately plunge-frozen into liquid ethane, kept at 93K in a temperature-controlled cryostat (33), using a Talmon-type manual plunger (35) in a 4 ${}^{{}^{\circ}}$C cold room.

#### GABA${}_{A}$ receptor.

The GABA${}_{\;}\text{A}$ receptor β3 homomer was expressed and purified as previously described (20, 36). Briefly, a construct encoding the human GABA${}_{\;}\text{A}$ receptor β3 subunit (UniProt ID P28472) with a truncated M3-M4 loop and an N-terminal SBP tag was cloned into the pHL vector (37) and was transiently expressed in Expi293 human cells. Following collection by centrifugation, the cell pellets were resuspended in PBS, pH 7.4 supplemented with 1% (v/v) mammalian protease inhibitor cocktail (Sigma-Aldrich) and the membranes solubilised with 1% (w/v) lauryl maltose neopentyl glycol (LMNG) for 1 h. The insoluble material was removed by centrifugation followed by a 2-h incubation with streptavidin beads (Thermo Scientific) at 4 ${}^{{}^{\circ}}$C. The beads were washed with PBS pH 7.4, 0.1% (w/v) LMNG to remove impurities. The receptor was then reconstituted in nanodiscs as described previously (38) and eluted in 2.5 mM biotin, PBS pH 7.4.

Prior to grid plunging, 1 mM histamine, 100 μM etomidate, and 3 μM megabody-25 (39) were added to the sample, which was incubated for 1 h on ice. A 3.5 μL volume of the sample was applied to a freshly glow-discharged (PELCO easiGlow, 30 mA for 120 s) 0.6/1.0 UltrAuFoil grid (40), blotted for 4 s and plunge-frozen using a Leica EM GP2 plunger at 14 ${}^{{}^{\circ}}$C and 99% humidity.

#### Apoferritin.

*Mus musculus* heavy chain apoferritin was purified as previously described (41). The protein was used at 10.8 mg/mL concentration for preparing cryoEM grids (UltrAuFoil 1.2/1.3) (40).

#### Catalase.

Human erythrocyte catalase (Sigma C3556) was prepared on cryoEM grids using a previously optimised protocol with the addition of 3-(3-cholamidopropyl-dimethylammonio)-2-hydroxy-1-propanesulfonate (CHAPSO, Sigma C3649) before plunge freezing (42). Grids (Quantifoil R1.2/1.3 300 mesh carbon on copper) were glow discharged for 30 s, and plunge-frozen at 100% humidity in a 4 ${}^{{}^{\circ}}$C cold room using a controlled environment plunging system of the Talmon type (35) with a 10 s blot time.

#### AHIR.

Acetohydroxy acid isomeroreductase (AHIR) from *Thermus thermophilus* was engineered to remove and mutate N and C terminal residues to improve protein stability and add a strep tag flanked by glycine linker residues at position 134–162. This construct was cloned into a pET28 bacterial expression vector and transformed into BL21 (DE3) *E. coli* cells for protein expression. A single colony was used to start a 10 mL overnight culture in LB with 1% glucose supplemented with kanamycin. The entire overnight culture was then used to inoculate 1 L of TB-AIM supplemented with kanamycin and grown at 37 ${}^{{}^{\circ}}$C for 3 h with 250 rpm shaking, before dropping the temperature to 20 ${}^{{}^{\circ}}$C for a further 20 h of growth. Cells were harvested by centrifugation at 4,000×g for 15 min, before resuspending in 5 mL/g of pellet in lysis buffer (20 mM HEPES, 500 mM NaCl, 3 mM imidazole, and 0.5 mM TCEP, pH 7.5) supplemented with 10 mg/mL Triton X-100, 0.5 mg/mL of lysozyme, 0.01 mg/mL benzonase, and 0.02 mg/mL of polymixin B. The resuspended cell pellet was lysed by snap freezing in liquid nitrogen then immediately thawing at room temperature before heating at 50 ${}^{{}^{\circ}}$C for 1 h, followed by centrifugation at 5,000 × g for 1 h to remove unwanted *E. coli* proteins. The remaining purification steps were carried out at room temperature. The supernatant was collected and applied to 10 mL of Ni-Sepharose FF resin under gravity flow, taking advantage of naturally occurring histidine residues on the AHIR N-terminus for affinity purification. The resin was washed with 100 mL of binding buffer (20 mM HEPES, 500 mM NaCl, 3 mM imidazole, and 0.5 mM TCEP, pH 7.5) before eluting in 20 mM HEPES, 500 mM NaCl, 300 mM imidazole, and 0.5 mM TCEP, pH 7.5, in 5- x 7.5 mL fractions. The peak fraction identified by SDS-PAGE was applied to a HiLoad 16/600 Superose 6 prep grade column for size exclusion chromatography, using 20 mM HEPES, 500 mM NaCl, and 0.5 mM TCEP, pH 7.5, as the mobile phase, producing a final yield of around 100 mg/L of protein. For cryoEM sample preparation, the protein was diluted to produce a final concentration of 150 mM NaCl. For grid preparation Quantifoil Cu R1.2/1.3 200 mesh grids were glow discharged (PELCO easiGlow, 20 mA for 60 s) before applying a 2 μL droplet at a concentration of 0.5 mg/mL to the grid and then blotting for 2 s, 0 force at 4 ${}^{{}^{\circ}}$C, 100% humidity using a ThermoFisher Vitrobot, and plunge freezing in liquid ethane.

#### GAPDH, glutamine synthetase, and aldehyde dehydrogenase.

Three test specimens—*Cryptosporidium parvum* glyceraldehyde-3-phosphate dehydrogenase (GAPDH), *E. coli* glutamine synthetase (GlnA), and *Homo sapiens* aldehyde dehydrogenase (ALDH1A1)—were prepared using the same procedure. Plasmids for bacterial expression containing the N-terminally 6×His-tagged proteins were obtained from Addgene: pET15-GAPDH–from C. Arrowsmith (Addgene 25125), pET28a-GlnA with a Y397F mutation was a gift from T. J. Kappock (Addgene 72391), and pDEST17-ALDH1A1–from R. Sobol (Addgene 189749) (43). The plasmids were transformed into BL21(DE3) pLysS cells. Overnight cultures, started from a single colony, were used to inoculate 2 L of TB (supplemented with the appropriate antibiotic) at a ratio of 1:200. The cultures were grown at 37 ${}^{{}^{\circ}}$C and 200 rpm until reaching an OD at 600 nm of 1.5 and then induced with 1 mM IPTG for 20 h at 200 rpm, 20 ${}^{{}^{\circ}}$C. Following collection by centrifugation, the cell pellets were resuspended in 200 mL lysis buffer (20 mM Tris-HCl pH 8, 300 mM NaCl), supplemented with protease inhibitor (cOmplete EDTA-free tablets, Roche), lysozyme (1 mg/mL, Sigma), and 4 μL benzonase nuclease (Merck). Cells were disrupted using a high-pressure homogeniser at 38,000 psi and 4 ${}^{{}^{\circ}}$C. Cell debris was removed by centrifugation at 30,000×g for 20 min at 4 ${}^{{}^{\circ}}$C, and the volume of the supernatants was adjusted to 350 mL each by adding lysis buffer. The cell lysates were then mixed with 2.5 mL Ni-NTA Agarose resin (Qiagen) and 0.5 mM TCEP. Binding to the resin was allowed to proceed for 2 h while shaking at 110 rpm at 10 ${}^{{}^{\circ}}$C. The beads were then collected by gravity flow and washed three times with 50 mL of 25 mM Tris-HCl pH 8, 400 mM NaCl, and 0.5 mM TCEP, containing increasing imidazole concentrations: 25 mM, 37.5 mM, and 50 mM, respectively. The proteins were eluted in 20 mL of 25 mM Tris-HCl pH 8, 400 mM NaCl, 0.5 mM TCEP, and 500 mM imidazole. The total yield from 2 L of culture for each of the specimens was 16 mg for GAPDH, 6 mg for GlnA, and 42 mg for ALDH1A1. The elutions were concentrated to 6.6 mg/mL, 5.4 mg/mL, and 13.9 mg/mL, respectively, using 15-mL concentrators (Vivaspin) with 30-kDa molecular cutoff for GAPDH and 100-kDa cutoff for the other two specimens, before size-exclusion chromatography. For size-exclusion chromatography, the same column (Superose 6 10/300 GL, Cytiva), equilibrated with 20 mM HEPES pH 7.4, 200 mM NaCl, 0.5 mM TCEP) at 4 ${}^{{}^{\circ}}$C was used for all specimens. All specimens were centrifuged at 21,000×g immediately prior to size exclusion to remove any large aggregates. For ALDH1A1, only half of the concentrated elution was loaded onto the column. All three proteins were eluted at 0.5 mL/min at 4 ${}^{{}^{\circ}}$C and the elutions collected in 0.8 mL fractions in three separate runs. For GAPDH and GlnA, the peak fractions were pooled and concentrated to 5 mg/mL and 4.1 mg/mL, respectively, using 4 mL concentrators (Vivaspin) with molecular weight cutoffs of 30 kDa and 100 kDa, respectively. For ALDH1A1, the concentration of the central peak fraction was sufficiently high (6.6 mg/mL), and it was used directly for preparing cryoEM grids. All specimens were plunge-frozen using a manual plunger of the Talmon type (35) in a 4 ${}^{{}^{\circ}}$C cold room, equilibrated at ≥90% relative humidity. For each specimen, a 3 μL droplet was applied to an UltrAuFoil 0.6/1.0 grid (precleaned with 180 s of 9:1 Ar:O${}_{2}$ plasma at 42 W power, 30 sccm) and blotted with filter paper (Whatman No. 1) from the foil side only for 14 s. The grid was then immediately plunged into liquid ethane held at 93 K in a temperature-controlled cryostat (33).

#### PaaZ.

Phenylacetic acid enzyme Z (PaaZ) from *E. coli* was purified as previously described (44). Graphene-oxide coated grids were prepared as described previously (45), with some modifications as described in ref. 44. PaaZ at 0.015 mg/mL concentration was applied to the graphene-oxide coated UltrAuFoil 1.2/1.3 grid, blotted with the Vitrobot for 3 s, and plunge-frozen into liquid ethane.

#### Lumazine synthase.

Lumazine synthase from *Aquifex aeolicus* (AaLS) was purified as previously described (46) and used at a concentration of 6.6 mg/mL to prepare cryoEM specimens. Grids (home-made HexAuFoil 0.3/0.3 gold on hexagonal gold) were glow discharged for 30 s, and plunge-frozen at 100% humidity in a 4 ${}^{{}^{\circ}}$C cold room using a controlled environment plunging system of the Talmon type (35) with a 10 s blot time. All 402 images were recorded from a single grid hexagon.

### Data Collection.

Micrographs were acquired manually using an in-house created low-dose interface to the detector and microscope. Datasets for each specimen were acquired using a side-entry liquid nitrogen-cooled specimen holder (Gatan 626) over a period of time from 4 to 12 h during a single day. Specific data collection settings are described for each specimen in their respective sections of the methods and in *SI Appendix*, Table S1.

### Data Processing.

All cryoEM datasets were processed using a similar approach. Briefly, the raw detector frames were converted into mrcs stacks (movies) with the appropriate number of fractions using in-house software. The details of how the raw detector frames were converted into image stacks follow the procedure described in ref. 17, with extensions to handle overlapping events, hot pixels, and the gap between the two detector modules. The movies were imported into RELION 4.0 (47) for motion correction, CTF estimation using CTFFIND-4.0 (10), and particle picking. Particles were either picked manually, or using the Laplacian-of-Gaussian picker, or using 2D templated generated from 2D classes after manual picking. Particles were initially extracted in small boxes (≈4/3× the size of the particle) and subjected to 2D classification in RELION 4.0 or in cryoSPARC 4.1 (48). Initial models were obtained from the EMDB or calculated de novo from selected 2D classes in cryoSPARC. Particle sets were pruned further by heterogeneous refinement in cryoSPARC or 3D classification in RELION, or a combination of both. The final particle sets were refined in RELION, with multiple rounds of CTF refinement to calculate the beam tilt, anisotropic magnification, higher-order aberrations, per-particle defocus and per-micrograph astigmatism, and Bayesian polishing (11), which was also used to increase the particle box sizes sufficiently to capture delocalised information set by the defocus value (49).

### Model Building and Map-model FSC Calculations.

Small errors in the pixel size of maps were corrected by comparison to high-resolution PDB models using the fit in map function in Chimera (50). The atomic models were located using MOLREP (51) and rigid-body refined using Coot’s jiggle-fit function (52). Finally, the coordinates and atomic displacement parameters in the asymmetric units were refined against unsharpened half maps with jelly-body restraints and point group symmetry constraints using Servalcat (23). Map-model FSC values were calculated using symmetry-expanded models and full maps, with the masks calculated from the models. The PDB codes for the initial models used for building were as follows (structure: initial model): DPS: 6ZGL, 70S: 7K00, GABAAR: 7A5V, apoF: 7A4M, catalase: 7P8W, AHIR: 8PRU, GAPDH: 3CPS, GlnA: 7W85, ALDH1A1: 4WJ9, PaaZ: 6JQL, and AaLS: 1HQK.

## Supplementary Material

Appendix 01 (PDF)

## Data Availability

All datasets used for structure determination are publicly available in EMPIAR (EMPIAR-11752), the eleven reconstructed maps in the EMDB (EMD-17958–EMD-17968), and the eleven refined atomic model coordinates in the PDB (PDB-8PV9–PDB-8PVJ). The code used for converting electron event files to MRC format is available from the corresponding author’s website (www.mrc-lmb.cam.ac.uk/crusso) and is deposited in ZENODO (53).
