# Supplementary material for: Structure determination by cryoEM at 100 keV
Source: Proc Natl Acad Sci U S A. 2023 Nov 27;120(49):e2312905120. doi: 10.1073/pnas.2312905120 (PMC10710074; doi:10.1073/pnas.2312905120)
Supplement: Supplementary file 1 — Appendix 01 (PDF) [file pnas.2312905120.sapp.pdf]

Supporting Information for

Structure determination by cryoEM at 100 keV

Greg McMullan, Katerina Naydenova, Daniel Mihaylov, Keitaro Yamashita, Mathew J. Peet, Hugh Wilson, Joshua L. Dickerson, Shaoxia Chen, Giuseppe Cannone, Yang Lee, Katherine A. Hutchings, Olivia Gittins, Mohamed Sobhy, Torquil Wells, Mohamed M. El-Gomati, Jason Dalby, Matthias Meffert, Clemens Schulze-Briese, Richard Henderson, Christopher J. Russo

Christopher J. Russo
E-mail: crusso@mrc-lmb.cam.ac.uk

This PDF file includes:

Figs. S1 to S14
Table S1
SI References

	DPS	70S ribosome	$GABA_A$ receptor	Apoferritin	Catalase	AHIR	GAPDH	GlnA	ALDH1A1	PaaZ	AaLS
Specimen support	UltrAuFoil	UltrAuFoil	UltrAuFoil	UltrAuFoil	QF R1.2/1.3	QF R1.2/1.3	UltrAuFoil	UltrAuFoil	UltrAuFoil	UltrAuFoil	HexAuFoil
	0.6/1.0	0.6/1.0	0.6/1.0	1.2/1.3	300M C on Cu	200M C on Cu	0.6/1.0	0.6/1.0	0.6/1.0	1.2/1.3+GO	0.3/0.3
No. micrographs	342	450	398	429	484	419	429	408	416	331	402
No. picked particles	94,424	26,054	14,484	61,153	59,611	29,199	146,382	45,890	113,604	45,382	16458
No. particles in reconstruction	48,896	11,872	5,706	26,745	27,165	15,220	19,411	9,881	32,968	23,716	8565
Nominal magnification	250,000×	200,000×	250,000×	250,000×	250,000×	250,000×	250,000×	250,000×	250,000×	250,000×	250,000×
Pixel size	1.724 Å/pix	2.062 Å/pix	1.656 Å/pix	1.676 Å/pix	1.651 Å/pix	1.663 Å/pix	1.683 Å/pix	1.692 Å/pix	1.683 Å/pix	1.655 Å/pix	1.657 Å/px
Defocus range	$0.2-1.4$ µm	$0.2-1.5$ µm	$0.3-1.1$ µm	$0.1-1.9$ µm	$0.2-1.5$ µm	$0.3-1.4$ µm	$0.2-1.1$ µm	$0.1-1.4$ µm	$0.1-1.4$ µm	$0.5-2.0$ µm	0.2–1.0 µm
Mean defocus	0.7 µm	0.7 µm	0.8 µm	0.5 µm	0.8 µm	0.9 µm	0.6 µm	0.7 µm	0.7 µm	1.3 µm	0.6 µm
Number of raw frames	16,384	16,384	32,768	32,768	32,768	32,768	16,384	16,384	16,384	32,768	16,384
El. fluence per raw frame	1/150 e^-/pix/fr	1/158 e^-/pix/fr	1/151 e^-/pix/fr	1/150 e^-/pix/fr	1/265 e^-/pix/fr	1/155 e^-/pix/fr	1/151 e^-/pix/fr	1/152 e^-/pix/fr	1/152 e^-/pix/fr	1/150 e^-/pix/fr	1/149 e^-/px/fr
Total electron fluence	40.0 e^-/Å^2	24.4 e^-/Å^2	79.3 e^-/Å^2	80.0 e^-/Å^2	45.4 e^-/Å^2	79.6 e^-/Å^2	39.8 e^-/Å^2	40.6 e^-/Å^2	40.6 e^-/Å^2	80.0 e^-/Å^2	40.3 e^-/Å^2
No. frames after grouping	64	32	128	64	64	90	64	64	64	50	64
El. fluence per grouped frame	0.63 e^-/Å^2/fr	0.76 e^-/Å^2/fr	0.62 e^-/Å^2/fr	1.25 e^-/Å^2/fr	0.71 e^-/Å^2/fr	0.88 e^-/Å^2/fr	0.62 e^-/Å^2/fr	0.63 e^-/Å^2/fr	0.63 e^-/Å^2/fr	1.60 e^-/Å^2/fr	0.63 e^-/Å^2/fr
Resolution (0.143 FSC, masked)	2.7 Å	4.5 Å	3.2 Å	2.6 Å	3.4 Å	3.3 Å	2.9 Å	3.4 Å	2.9 Å	3.7 Å	3.0 Å
Efficiency of orient. distrib.	0.9	0.7	0.8	0.9	0.9	0.9	0.9	0.7	0.8	0.8	0.9
Symmetry imposed	T	C1	C5	O	D2	T	D2	D6	D2	D3	I
**Model composition in asymmetric unit**											
peptide residues	154	5587	455	172	502	317	337	435	493	678	155
RNA residues	0	4555	0	0	0	0	0	0	0	0	0
peptide atoms	1217	43768	3672	1409	4037	2448	2491	3394	3792	5116	1181
RNA atoms	0	97795	0	0	0	0	0	0	0	0	0
other atoms	0	2	173	2	91	0	44	0	1	0	0
**Average B-factors (Å^2)**											
peptide	109.6	126.0	104.0	100.6	106.5	130.9	100.3	158.6	90.5	145.2	117.6
RNA		115.8									
others		143.7	95.3	115.5	67.3						
**R.m.s. deviation**											
bond lengths (Å)	0.0075	0.0061	0.0084	0.0100	0.0082	0.0078	0.0074	0.0066	0.0076	0.0077	0.0080
bond angles (°)	1.55	1.35	1.65	1.82	1.71	1.69	1.76	1.48	1.66	1.53	1.75
**Validation**											
Clashscore	3.43	1.61	1.17	1.61	0.43	1.25	0.60	5.81	0.56	1.37	1.52
Rotamer outliers (%)	2.35	3.59	1.27	0.00	1.39	3.96	1.12	1.10	1.49	1.13	1.67
**Ramachandran**											
favored (%)	99.34	96.22	98.44	98.24	96.00	97.12	95.52	95.36	97.76	98.37	98.69
allowed (%)	0.66	3.60	1.56	1.76	3.80	2.88	4.18	4.64	2.04	1.48	1.31
outliers (%)	0.00	0.18	0.00	0.00	0.20	0.00	0.30	0.00	0.20	0.15	0.00
PDB code	8PV9	8PVA	8PVB	8PVC	8PVD	8PVE	8PVF	8PVG	8PVH	8PVI	8PVJ
EMDB code	17958	17959	17960	17961	17962	17963	17964	17965	17966	17967	17968

Table S1. CryoEM data collection and processing. Summary of the cryoEM data collection conditions and data processing results for the example specimens imaged at 100 keV in this work.

Supplementary Figures

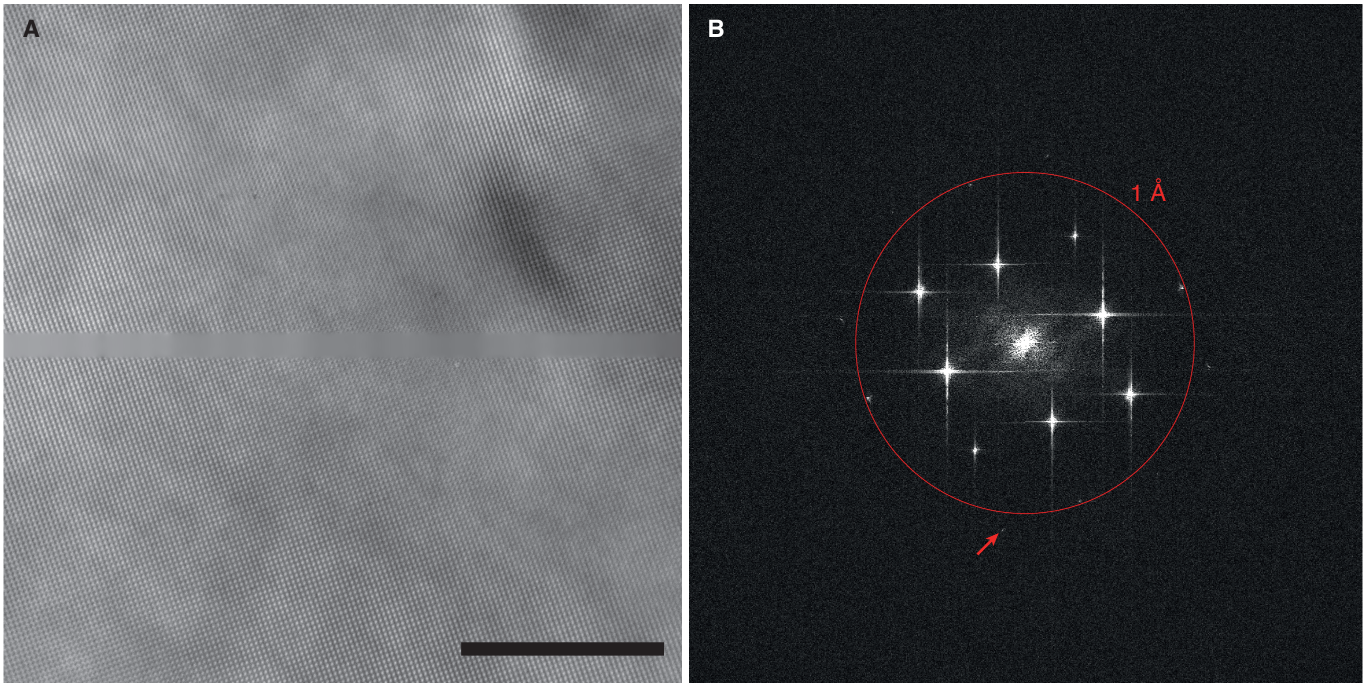

Fig. S1. A $\langle 200 \rangle$ oriented gold foil imaged at 1.5 M$\times$ magnification A micrograph (**A**) and the amplitude of the FFT (**B**) are shown. Scale bar is 100 Å. Refections at 2.04 Å, 1.4 Å, 1.02 Å, 0.9 Å are visible. The $\langle 420 \rangle$ reflection is indicated with an arrow and the $1/1$ Å^{-1} circle is overlaid in red.

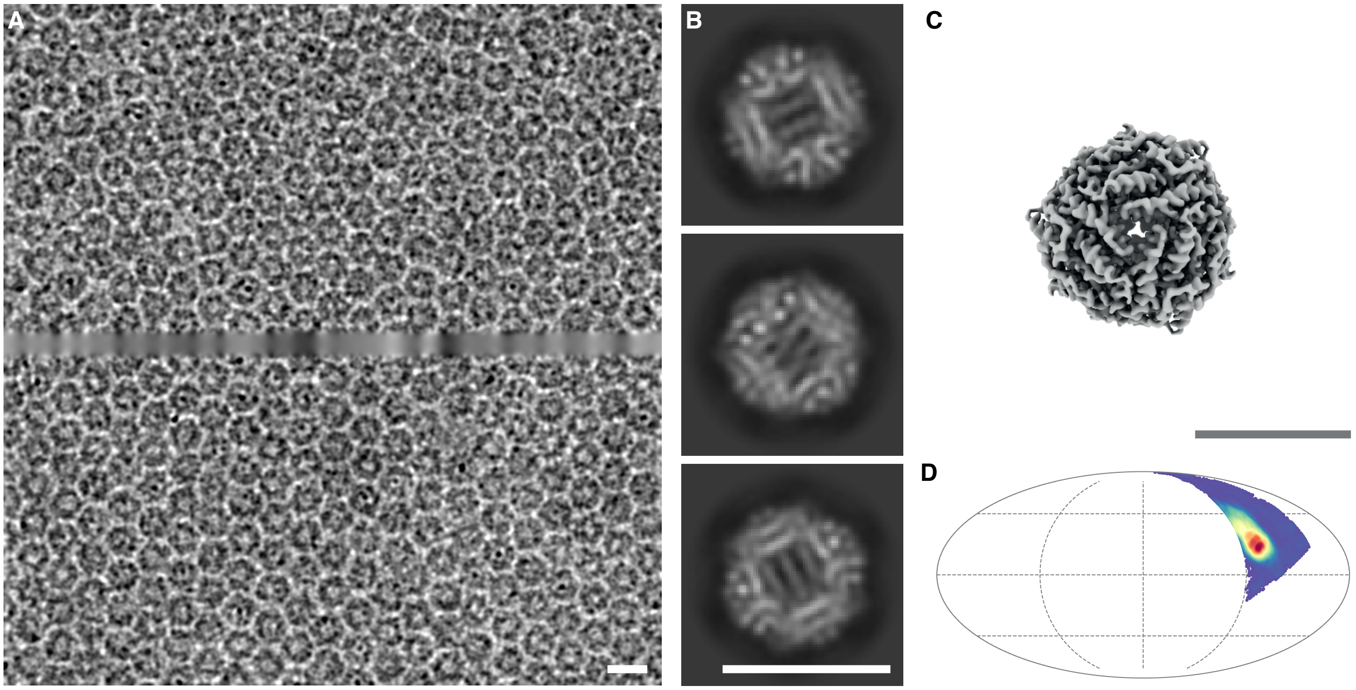

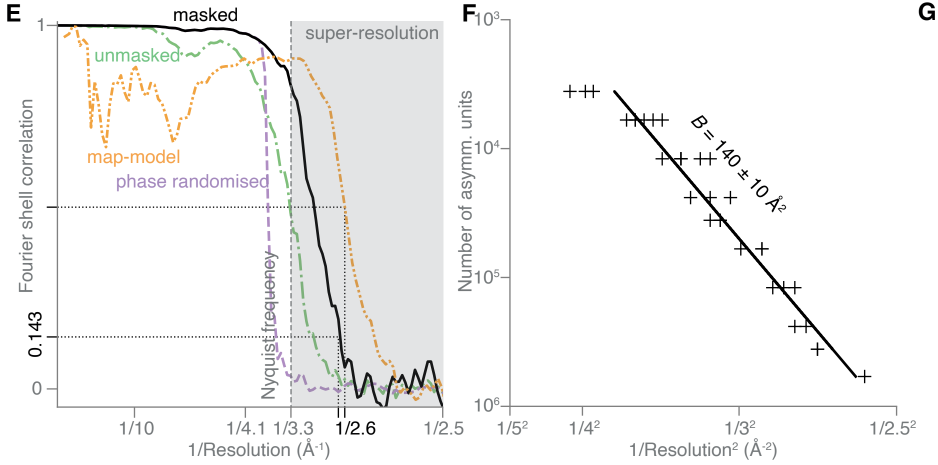

G

Number of 1k × 1k micrographs	360
Number of picked particles	94,424
Number of final particles	48,896
Nominal maginfication	250,000×
Pixel size (phys. & super-res.)	1.724 Å, 0.862 Å
Defocus range	0.2 - 1.4 µm
Average defocus	0.7 µm
Exposure time	3.64 s
Number of raw frames	16,384
Fluence per raw frame	1/150 e⁻/pix/fr
Total electron fluence	40.0 e⁻/Å²
Frames after grouping	64
Fluence per grouped frame	0.63 e⁻/Å²/fr
Resolution (FSC = 0.143)	2.7 Å
Efficiency of OD	0.9
Rosenthal *B*-factor	140 Å²
Symmetry imposed	T
PDB code	8PV9
EMDB code	17958

Fig. S2. Structure determination for DPS A representative micrograph (**A**), and three selected reference free 2D class averages (**B**) are shown. Scale bars are all 100 Å. The final reconstructed map is shown in **C**, and a Mollewiede equal area plot of the particle orientation distribution on the sphere is shown in **D**. Fourier shell correlation (FSC) plots (**E**) for the two independently refined, unmasked, dose-weighted, half-maps (green dash-dotted curve), phase-randomised dose-weighted half maps (purple dashed curve), the final independently refined, masked, dose-weighted, half-maps (black solid curve), and the map-model FSC after model refinement (orange curve) are shown. A Rosenthal plot (1) of the resolution vs. number of particles is shown in **F**, and a table of the data collection and analysis statistics is shown in **G**.

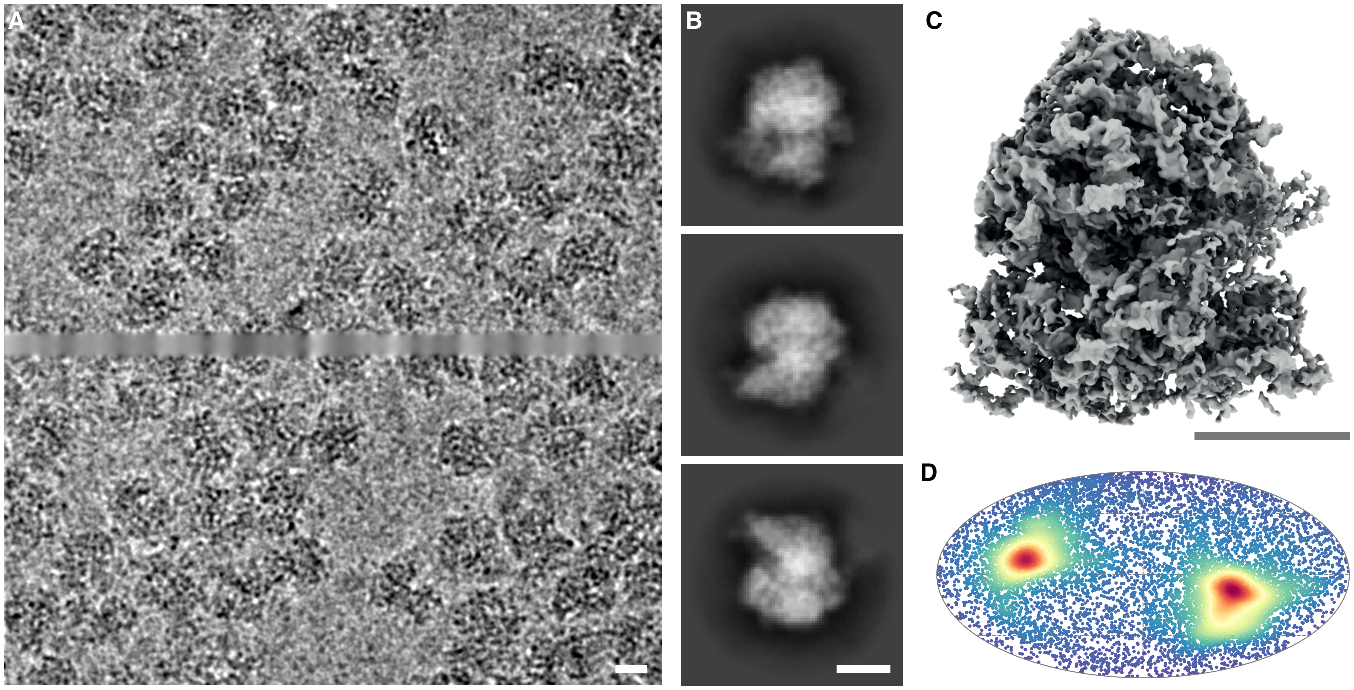

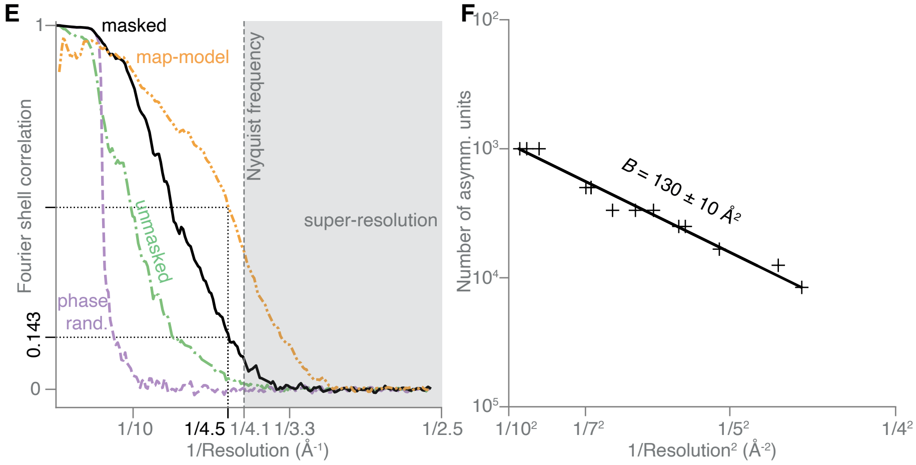

G

Number of 1k × 1k micrographs	450
Number of picked particles	26,054
Number of final particles	11,872
Nominal maginfication	200,000×
Pixel size (phys. & super-res.)	2.062 Å, 1.031 Å
Defocus range	0.2 - 1.5 µm
Average defocus	0.7 µm
Exposure time	3.64 s
Number of raw frames	16,384
Fluence per raw frame	1/158 e⁻/pix/fr
Total electron fluence	24.4 e⁻/Å²
Frames after grouping	32
Fluence per grouped frame	0.76 e⁻/Å²/fr
Resolution (FSC = 0.143)	4.5 Å
Efficiency of OD	0.7
Rosenthal *B*-factor	130 Å²
Symmetry imposed	C1
PDB code	8PVA
EMDB code	17959

Fig. S3. Structure determination for E. coli 70S ribosome A representative micrograph (**A**), and three selected reference free 2D class averages (**B**) are shown. Scale bars are all 100 Å. The final reconstructed map is shown in **C**, and a Mollewiede equal area plot of the particle orientation distribution on the sphere is shown in **D**. Fourier shell correlation (FSC) plots (**E**) for the two independently refined, unmasked, dose-weighted, half-maps (green dash-dotted curve), phase-randomised dose-weighted half maps (purple dashed curve), the final independently refined, masked, dose-weighted, half-maps (black solid curve), and the map-model FSC after model refinement (orange curve) are shown. A Rosenthal plot (1) of the resolution vs. number of particles is shown in **F**, and a table of the data collection and analysis statistics is shown in **G**.

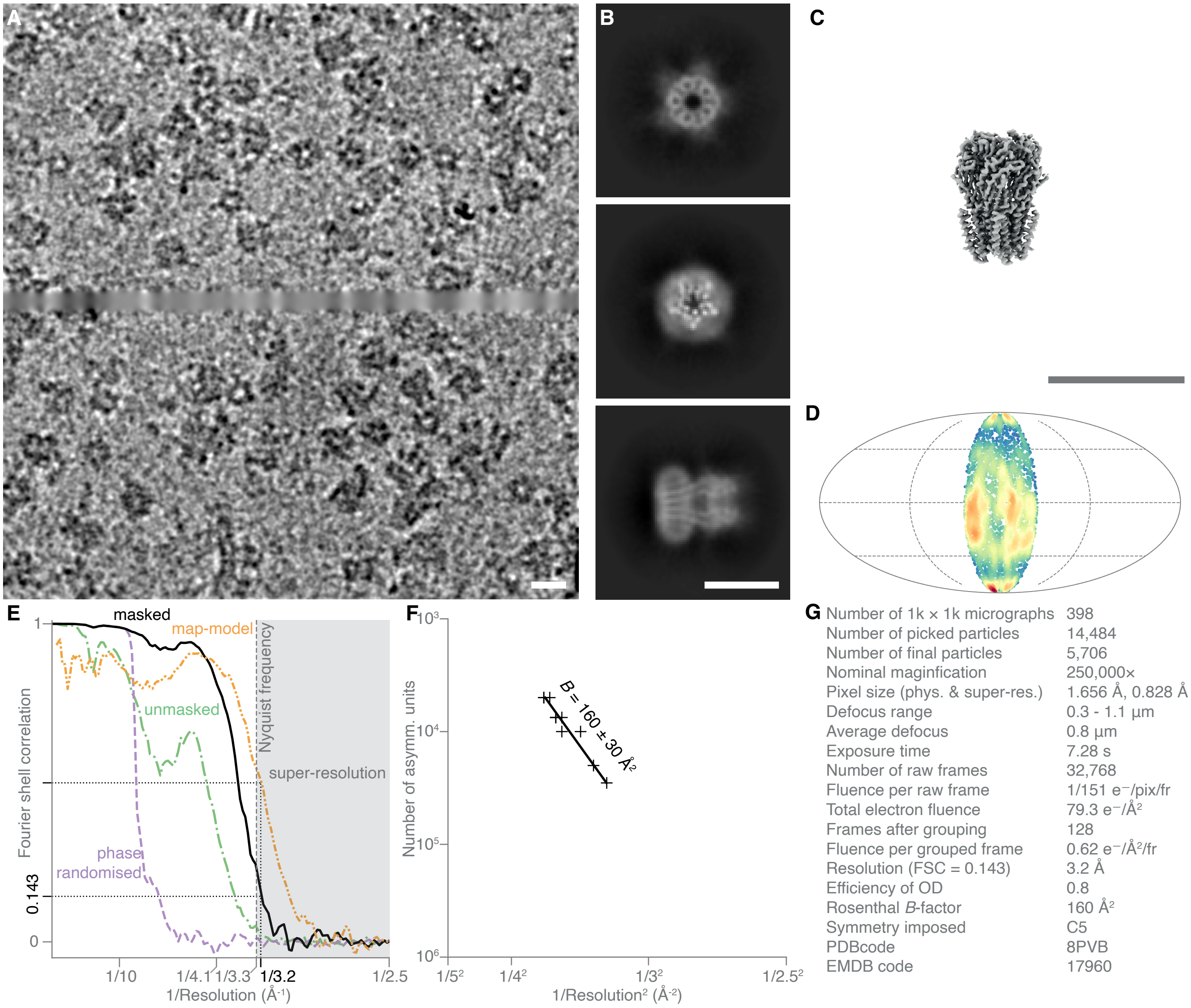

Number of 1k × 1k micrographs	398
Number of picked particles	14,484
Number of final particles	5,706
Nominal maginfication	250,000×
Pixel size (phys. & super-res.)	1.656 Å, 0.828 Å
Defocus range	0.3 - 1.1 µm
Average defocus	0.8 µm
Exposure time	7.28 s
Number of raw frames	32,768
Fluence per raw frame	1/151 e⁻/pix/fr
Total electron fluence	79.3 e⁻/Å²
Frames after grouping	128
Fluence per grouped frame	0.62 e⁻/Å²/fr
Resolution (FSC = 0.143)	3.2 Å
Efficiency of OD	0.8
Rosenthal *B*-factor	160 Å²
Symmetry imposed	C5
PDBcode	8PVB
EMDB code	17960

Fig. S4. Structure determination for GABA$_A$ receptor A representative micrograph (**A**), and three selected reference free 2D class averages (**B**) are shown. Scale bars are all 100 Å. The final reconstructed map is shown in **C**, and a Mollewiede equal area plot of the particle orientation distribution on the sphere is shown in **D**. Fourier shell correlation (FSC) plots (**E**) for the two independently refined, unmasked, dose-weighted, half-maps (green dash-dotted curve), phase-randomised dose-weighted half maps (purple dashed curve), the final independently refined, masked, dose-weighted, half-maps (black solid curve), and the map-model FSC after model refinement (orange curve) are shown. A Rosenthal plot (1) of the resolution vs. number of particles is shown in **F**, and a table of the data collection and analysis statistics is shown in **G**.

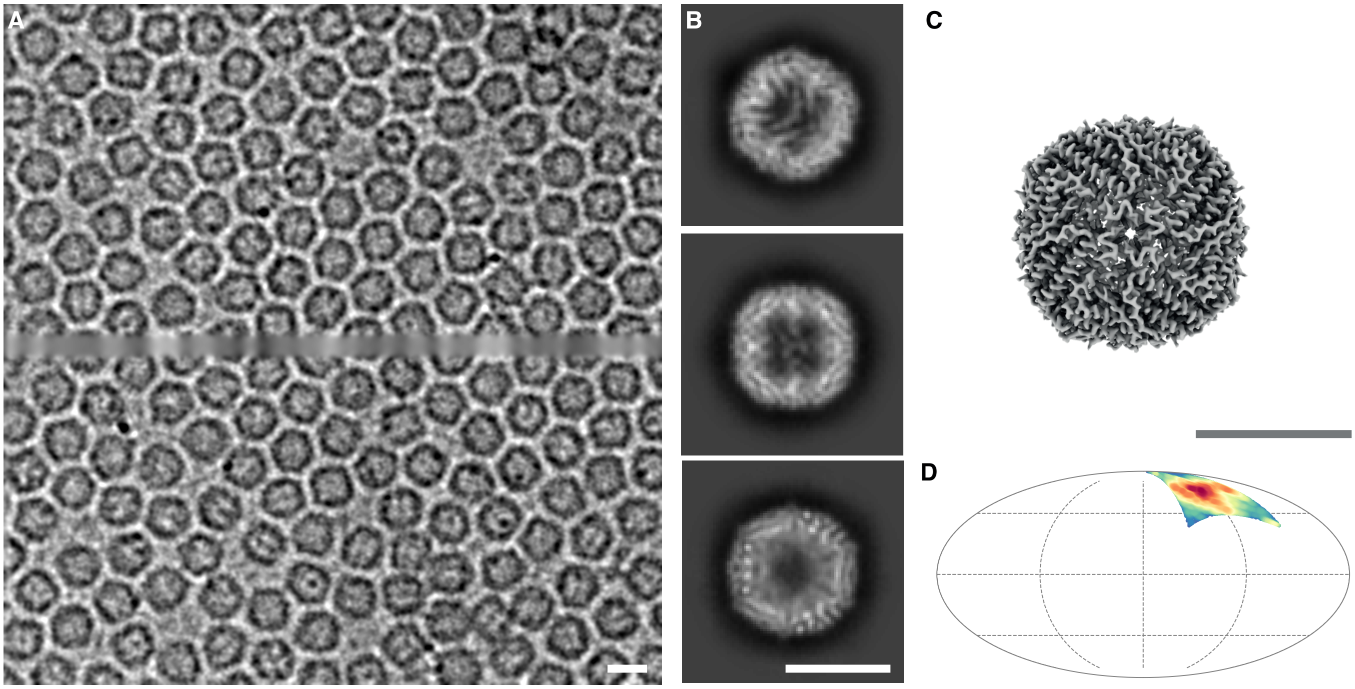

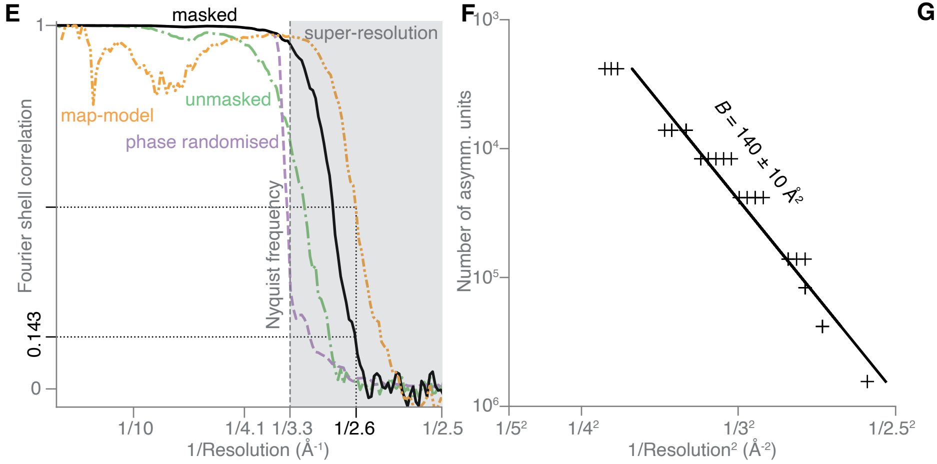

G

Number of 1k × 1k micrographs	429
Number of picked particles	61,153
Number of final particles	26,745
Nominal maginfication	250,000×
Pixel size (phys. & super-res.)	1.676 Å, 0.838 Å
Defocus range	0.1 - 1.9 µm
Average defocus	0.5 µm
Exposure time	7.28 s
Number of raw frames	32,768
Fluence per raw frame	1/150 e⁻/pix/fr
Total electron fluence	80.0 e⁻/Å²
Frames after grouping	64
Fluence per grouped frame	1.25 e⁻/Å²/fr
Resolution (FSC = 0.143)	2.6 Å
Efficiency of OD	0.9
Rosenthal *B*-factor	140 Å²
Symmetry imposed	O
PDB code	8PVC
EMDB code	17961

Fig. S5. Structure determination for apoferritin A representative micrograph (**A**), and three selected reference free 2D class averages (**B**) are shown. Scale bars are all 100 Å. The final reconstructed map is shown in **C**, and a Mollewiede equal area plot of the particle orientation distribution on the sphere is shown in **D**. Fourier shell correlation (FSC) plots (**E**) for the two independently refined, unmasked, dose-weighted, half-maps (green dash-dotted curve), phase-randomised dose-weighted half maps (purple dashed curve), the final independently refined, masked, dose-weighted, half-maps (black solid curve), and the map-model FSC after model refinement (orange curve) are shown. A Rosenthal plot (1) of the resolution vs. number of particles is shown in **F**, and a table of the data collection and analysis statistics is shown in **G**.

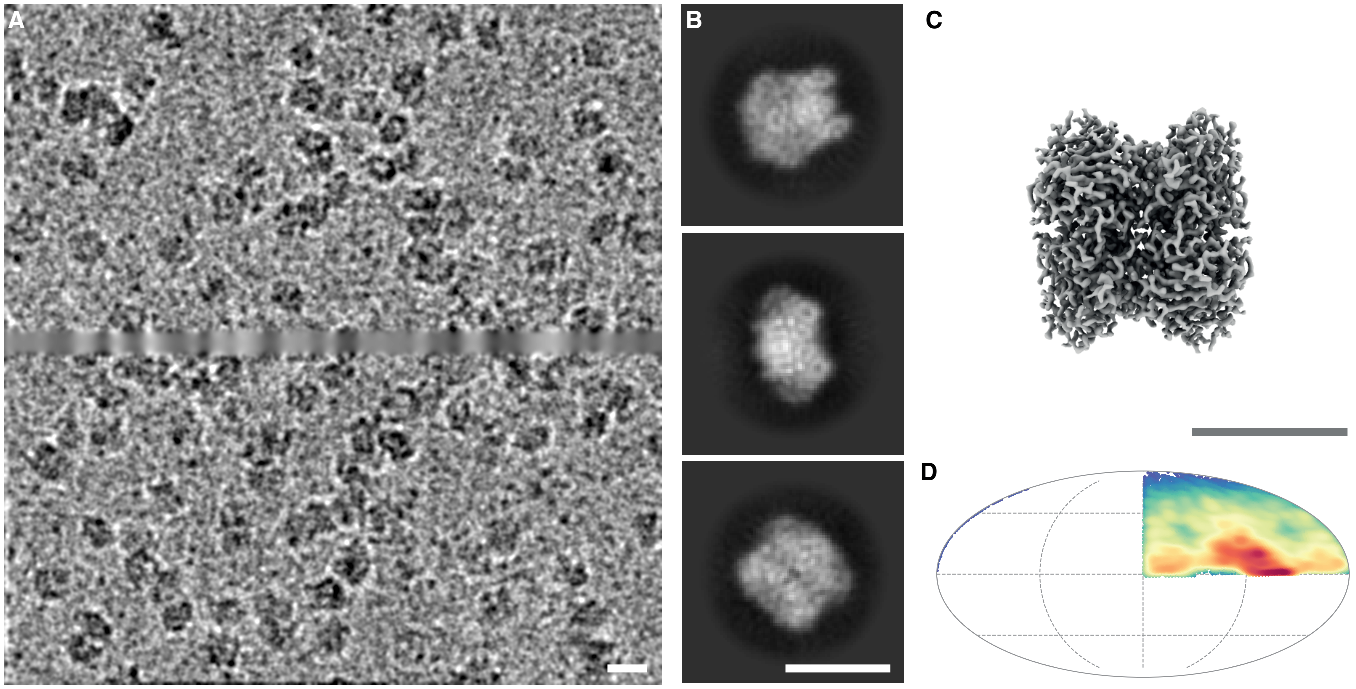

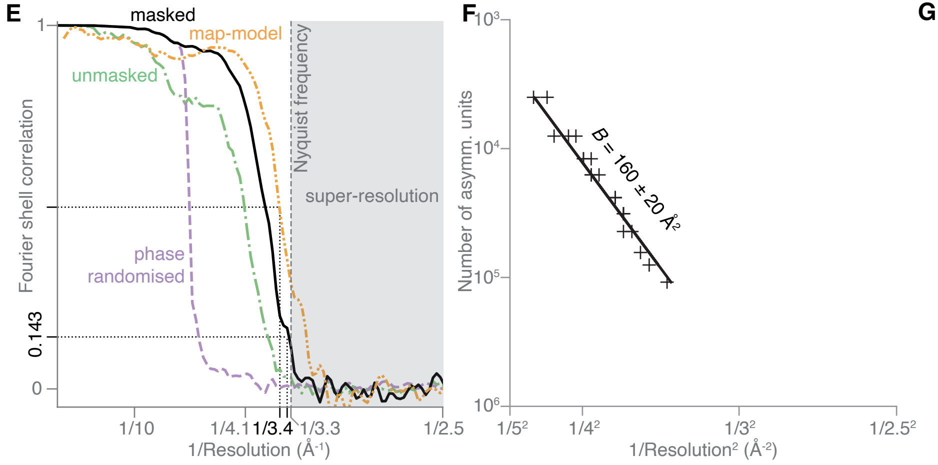

G

Number of 1k × 1k micrographs	484
Number of picked particles	59,611
Number of final particles	27,165
Nominal maginfication	250,000×
Pixel size (phys. & super-res.)	1.651 Å, 0.825 Å
Defocus range	0.2 - 1.5 µm
Average defocus	0.8 µm
Exposure time	7.28 s
Number of raw frames	32,768
Fluence per raw frame	1/265 e⁻/pix/fr
Total electron fluence	45.4 e⁻/Å²
Frames after grouping	64
Fluence per grouped frame	0.71 e⁻/Å²/fr
Resolution (FSC = 0.143)	3.4 Å
Efficiency of OD	0.9
Rosenthal *B*-factor	160 Å²
Symmetry imposed	D2
PDB code	8PVD
EMDB code	17962

Fig. S6. Structure determination for catalase A representative micrograph (**A**), and three selected reference free 2D class averages (**B**) are shown. Scale bars are all 100 Å. The final reconstructed map is shown in **C**, and a Mollewiede equal area plot of the particle orientation distribution on the sphere is shown in **D**. Fourier shell correlation (FSC) plots (**E**) for the two independently refined, unmasked, dose-weighted, half-maps (green dash-dotted curve), phase-randomised dose-weighted half maps (purple dashed curve), the final independently refined, masked, dose-weighted, half-maps (black solid curve), and the map-model FSC after model refinement (orange curve) are shown. A Rosenthal plot (1) of the resolution vs. number of particles is shown in **F**, and a table of the data collection and analysis statistics is shown in **G**.

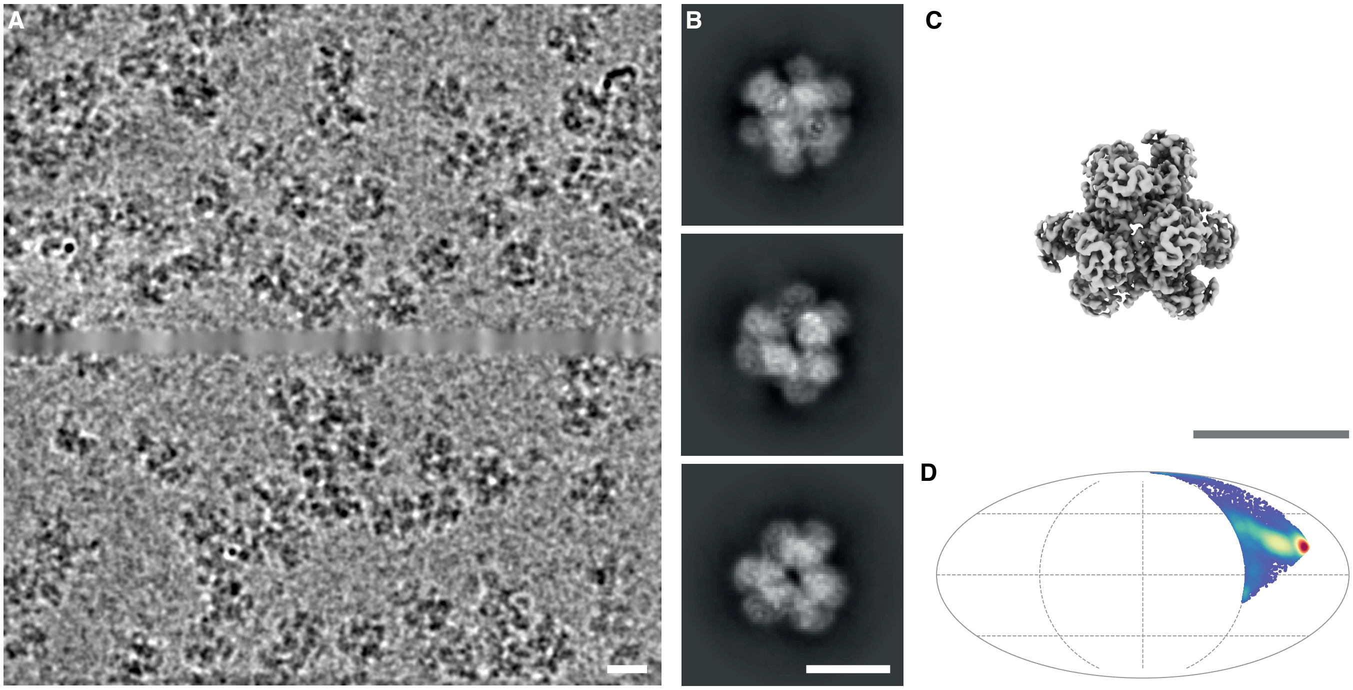

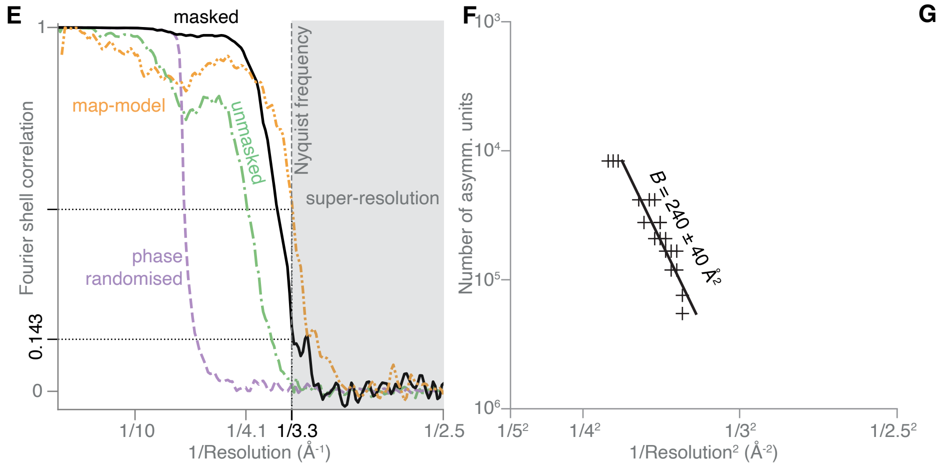

G

Number of 1k × 1k micrographs	419
Number of picked particles	29,119
Number of final particles	15,220
Nominal maginfication	250,000×
Pixel size (phys. & super-res.)	1.663 Å, 0.832 Å
Defocus range	0.3 - 1.4 µm
Average defocus	0.9 µm
Exposure time	7.28 s
Number of raw frames	32,768
Fluence per raw frame	1/155 e⁻/pix/fr
Total electron fluence	79.6 e⁻/Å²
Frames after grouping	90
Fluence per grouped frame	0.88 e⁻/Å²/fr
Resolution (FSC = 0.143)	3.3 Å
Efficiency of OD	0.9
Rosenthal *B*-factor	240 Å²
Symmetry imposed	T
PDB code	8PVE
EMDB code	17963

Fig. S7. Structure determination for AHIR A representative micrograph (**A**), and three selected reference free 2D class averages (**B**) are shown. Scale bars are all 100 Å. The final reconstructed map is shown in **C**, and a Mollewiede equal area plot of the particle orientation distribution on the sphere is shown in **D**. Fourier shell correlation (FSC) plots (**E**) for the two independently refined, unmasked, dose-weighted, half-maps (green dash-dotted curve), phase-randomised dose-weighted half maps (purple dashed curve), the final independently refined, masked, dose-weighted, half-maps (black solid curve), and the map-model FSC after model refinement (orange curve) are shown. A Rosenthal plot (1) of the resolution vs. number of particles is shown in **F**, and a table of the data collection and analysis statistics is shown in **G**.

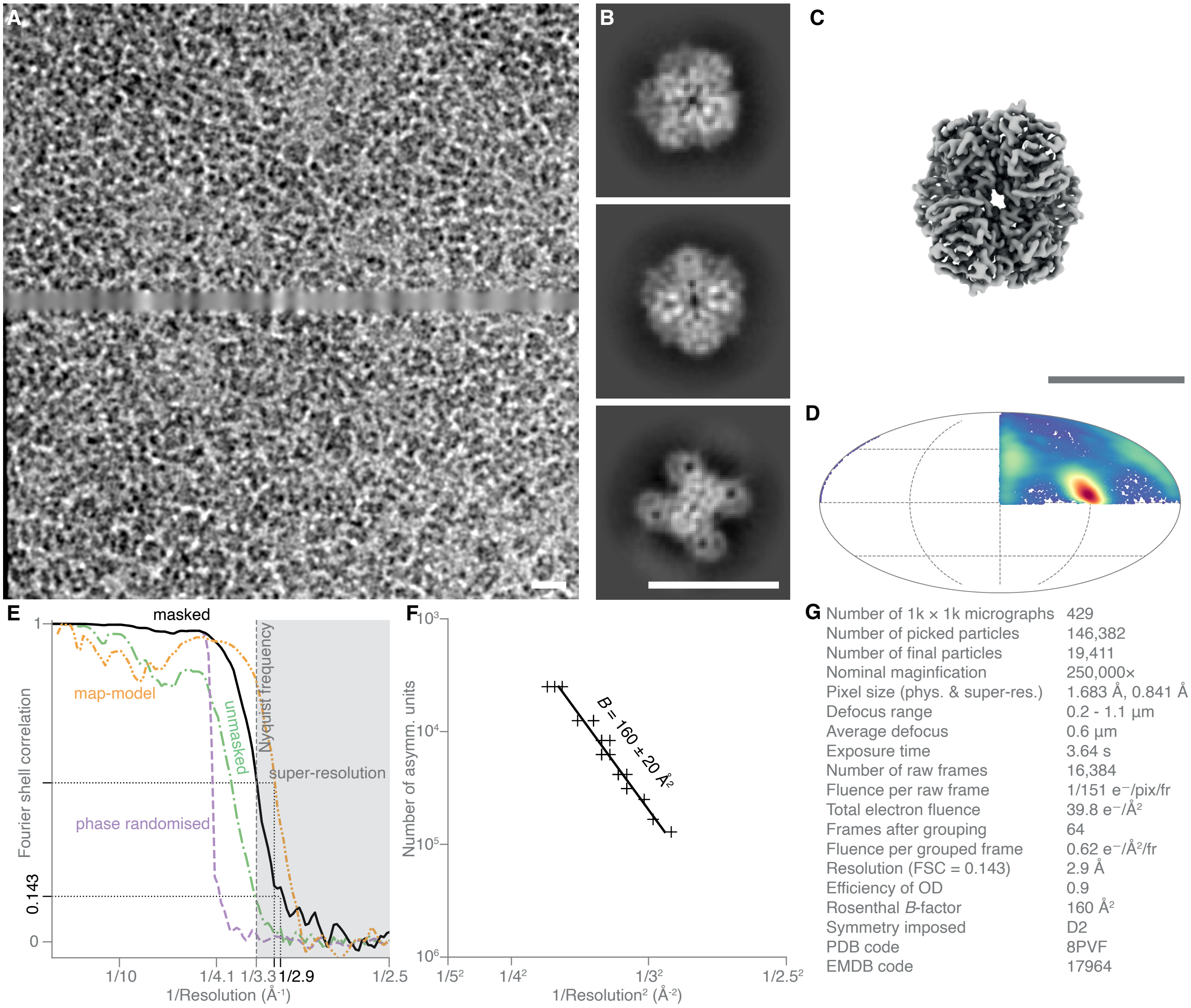

Number of 1k × 1k micrographs	429
Number of picked particles	146,382
Number of final particles	19,411
Nominal maginfication	250,000×
Pixel size (phys. & super-res.)	1.683 Å, 0.841 Å
Defocus range	0.2 - 1.1 µm
Average defocus	0.6 µm
Exposure time	3.64 s
Number of raw frames	16,384
Fluence per raw frame	1/151 e⁻/pix/fr
Total electron fluence	39.8 e⁻/Å²
Frames after grouping	64
Fluence per grouped frame	0.62 e⁻/Å²/fr
Resolution (FSC = 0.143)	2.9 Å
Efficiency of OD	0.9
Rosenthal *B*-factor	160 Å²
Symmetry imposed	D2
PDB code	8PVF
EMDB code	17964

Fig. S8. Structure determination for GAPDH A representative micrograph (**A**), and three selected reference free 2D class averages (**B**) are shown. Scale bars are all 100 Å. The final reconstructed map is shown in **C**, and a Mollewiede equal area plot of the particle orientation distribution on the sphere is shown in **D**. Fourier shell correlation (FSC) plots (**E**) for the two independently refined, unmasked, dose-weighted, half-maps (green dash-dotted curve), phase-randomised dose-weighted half maps (purple dashed curve), the final independently refined, masked, dose-weighted, half-maps (black solid curve), and the map-model FSC after model refinement (orange curve) are shown. A Rosenthal plot (1) of the resolution vs. number of particles is shown in **F**, and a table of the data collection and analysis statistics is shown in **G**.

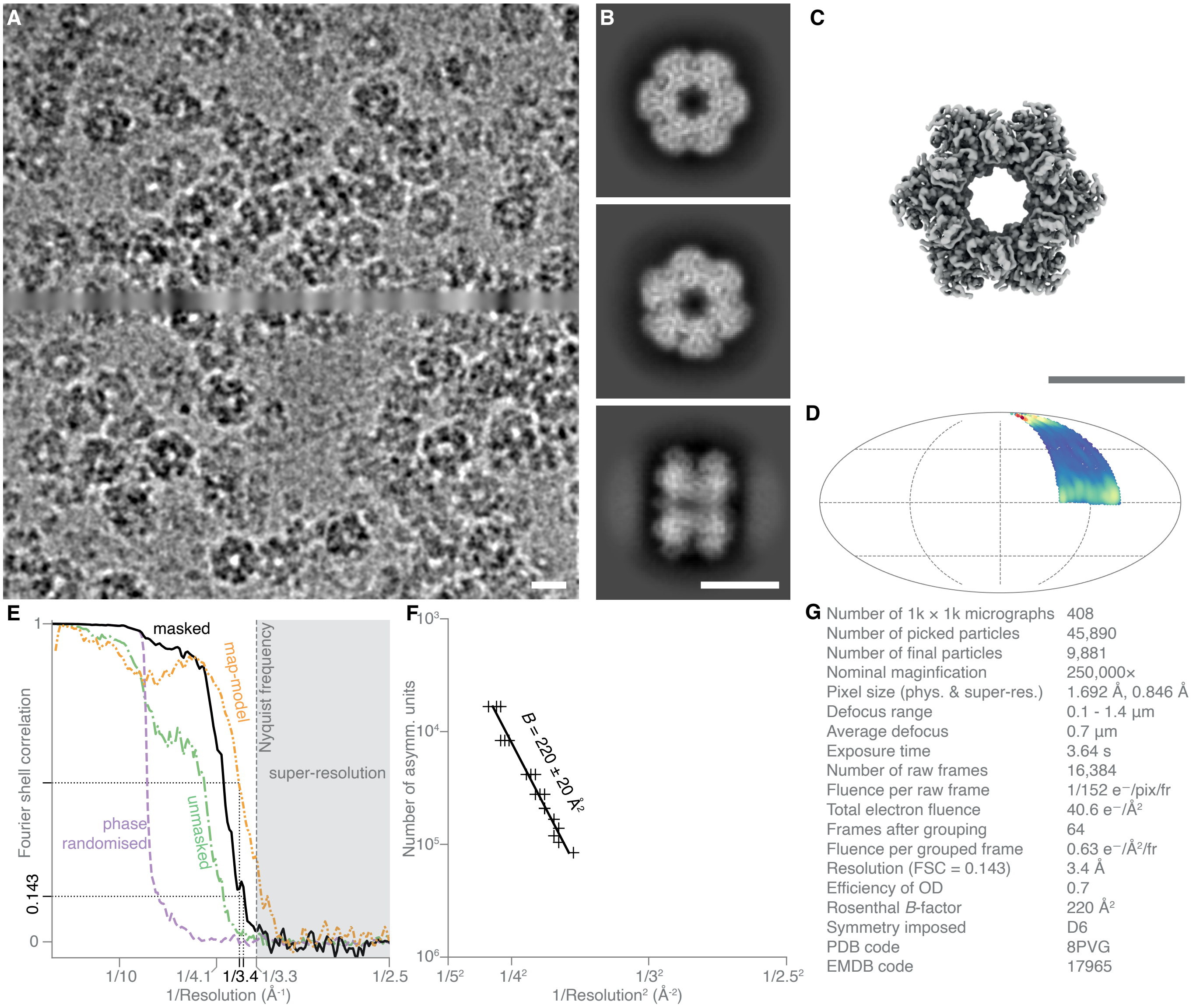

G

Number of 1k × 1k micrographs	408
Number of picked particles	45,890
Number of final particles	9,881
Nominal maginfication	250,000×
Pixel size (phys. & super-res.)	1.692 Å, 0.846 Å
Defocus range	0.1 - 1.4 µm
Average defocus	0.7 µm
Exposure time	3.64 s
Number of raw frames	16,384
Fluence per raw frame	1/152 e⁻/pix/fr
Total electron fluence	40.6 e⁻/Å²
Frames after grouping	64
Fluence per grouped frame	0.63 e⁻/Å²/fr
Resolution (FSC = 0.143)	3.4 Å
Efficiency of OD	0.7
Rosenthal *B*-factor	220 Å²
Symmetry imposed	D6
PDB code	8PVG
EMDB code	17965

Fig. S9. Structure determination for GlnA A representative micrograph (**A**), and three selected reference free 2D class averages (**B**) are shown. Scale bars are all 100 Å. The final reconstructed map is shown in **C**, and a Mollewiede equal area plot of the particle orientation distribution on the sphere is shown in **D**. Fourier shell correlation (FSC) plots (**E**) for the two independently refined, unmasked, dose-weighted, half-maps (green dash-dotted curve), phase-randomised dose-weighted half maps (purple dashed curve), the final independently refined, masked, dose-weighted, half-maps (black solid curve), and the map-model FSC after model refinement (orange curve) are shown. A Rosenthal plot (1) of the resolution vs. number of particles is shown in **F**, and a table of the data collection and analysis statistics is shown in **G**.

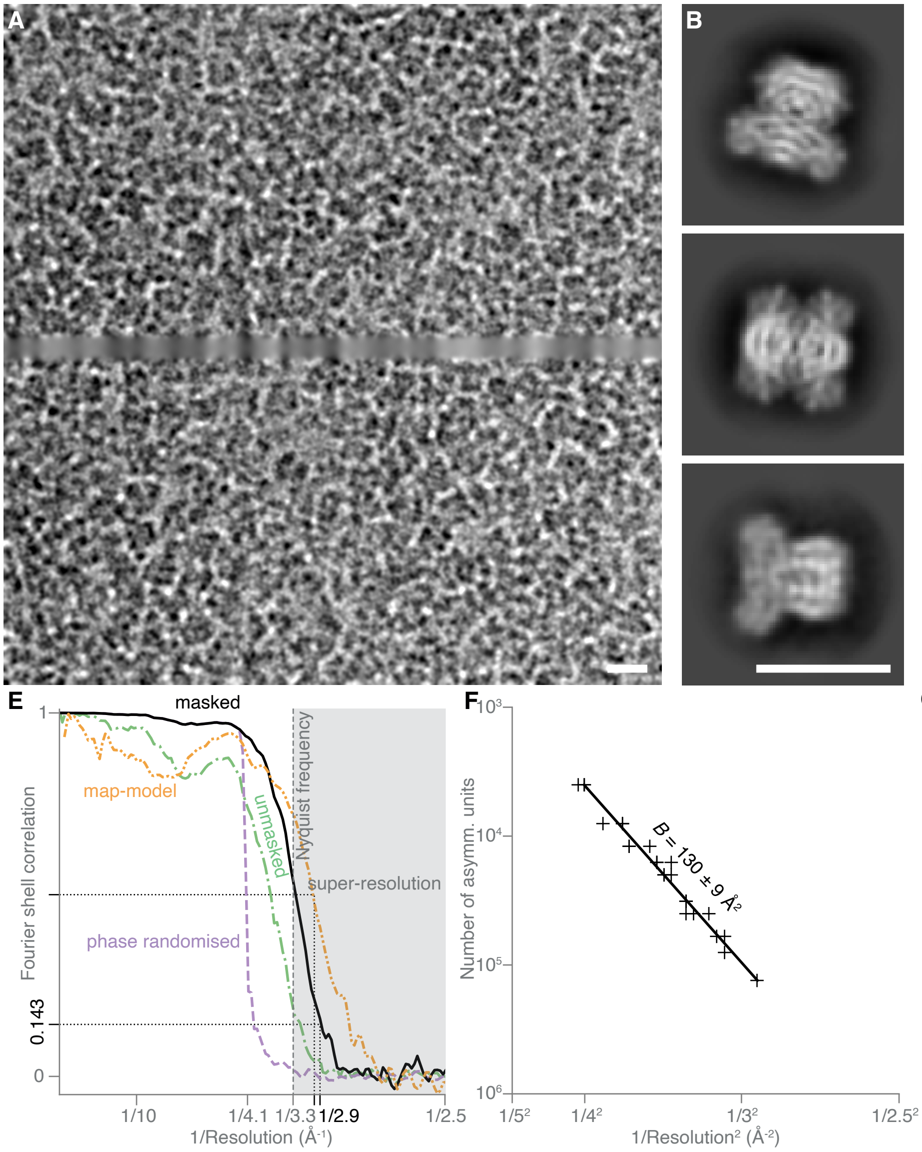

G

Number of 1k × 1k micrographs	416
Number of picked particles	113,604
Number of final particles	32,968
Nominal maginfication	250,000×
Pixel size (phys. & super-res.)	1.683 Å, 0.841 Å
Defocus range	0.1 - 1.4 µm
Average defocus	0.7 µm
Exposure time	3.64 s
Number of raw frames	16,384
Fluence per raw frame	1/152 e⁻/pix/fr
Total electron fluence	40.6 e⁻/Å²
Frames after grouping	64
Fluence per grouped frame	0.63 e⁻/Å²/fr
Resolution (FSC = 0.143)	2.9 Å
Efficiency of OD	0.8
Rosenthal *B*-factor	130 Å²
Symmetry imposed	D2
PDB code	8PVH
EMDB code	17966

Fig. S10. Structure determination for aldehyde dehydrogenase A representative micrograph (**A**), and three selected reference free 2D class averages (**B**) are shown. Scale bars are all 100 Å. The final reconstructed map is shown in **C**, and a Mollewiede equal area plot of the particle orientation distribution on the sphere is shown in **D**. Fourier shell correlation (FSC) plots (**E**) for the two independently refined, unmasked, dose-weighted, half-maps (green dash-dotted curve), phase-randomised dose-weighted half maps (purple dashed curve), the final independently refined, masked, dose-weighted, half-maps (black solid curve), and the map-model FSC after model refinement (orange curve) are shown. A Rosenthal plot (1) of the resolution vs. number of particles is shown in **F**, and a table of the data collection and analysis statistics is shown in **G**.

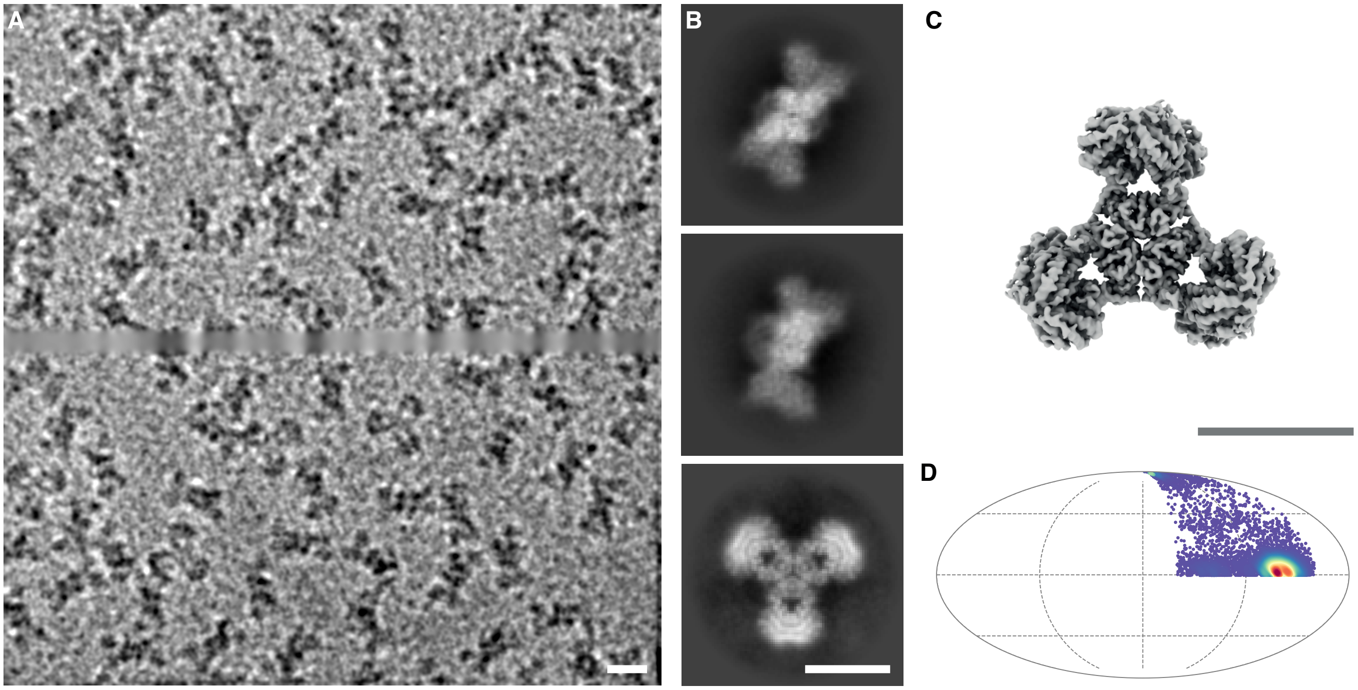

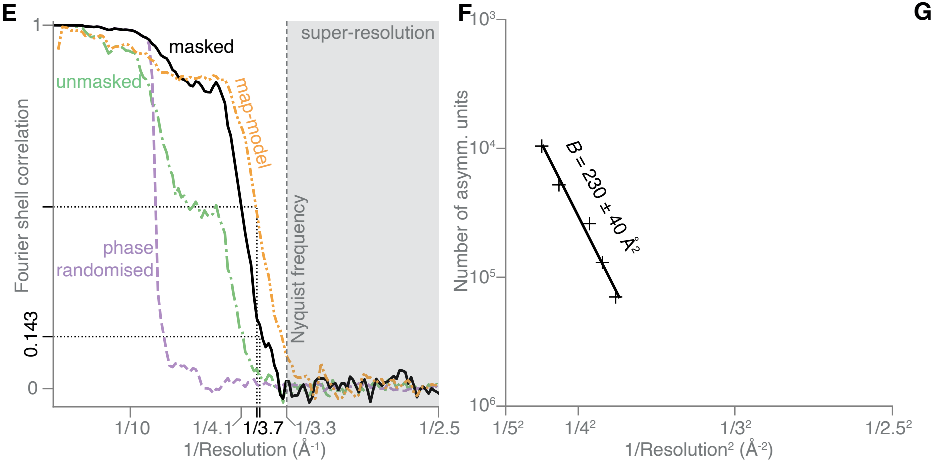

G

Number of 1k × 1k micrographs	331
Number of picked particles	45,382
Number of final particles	23,716
Nominal maginfication	250,000×
Pixel size (phys. & super-res.)	1.655 Å, 0.828 Å
Defocus range	0.5 - 2.0 µm
Average defocus	1.3 µm
Exposure time	7.28 s
Number of raw frames	32,768
Fluence per raw frame	1/150 e⁻/pix/fr
Total electron fluence	80.0 e⁻/Å²
Frames after grouping	50
Fluence per grouped frame	1.60 e⁻/Å²/fr
Resolution (FSC = 0.143)	3.7 Å
Efficiency of OD	0.8
Rosenthal *B*-factor	230 Å²
Symmetry imposed	D3
EMPIAR code	8PVI
EMDB code	17967

Fig. S11. Structure determination for PaaZ A representative micrograph (**A**), and three selected reference free 2D class averages (**B**) are shown. Scale bars are all 100 Å. The final reconstructed map is shown in **C**, and a Mollewiede equal area plot of the particle orientation distribution on the sphere is shown in **D**. Fourier shell correlation (FSC) plots (**E**) for the two independently refined, unmasked, dose-weighted, half-maps (green dash-dotted curve), phase-randomised dose-weighted half maps (purple dashed curve), the final independently refined, masked, dose-weighted, half-maps (black solid curve), and the map-model FSC after model refinement (orange curve) are shown. A Rosenthal plot (1) of the resolution vs. number of particles is shown in **F**, and a table of the data collection and analysis statistics is shown in **G**.

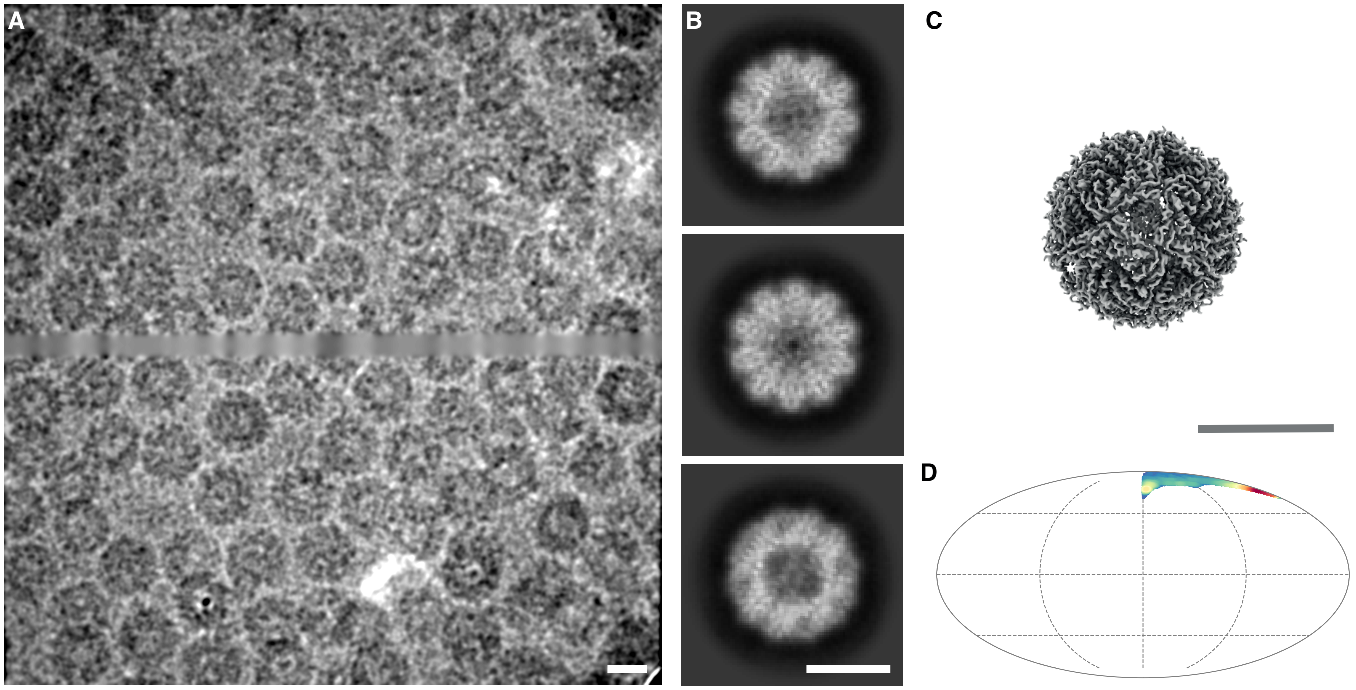

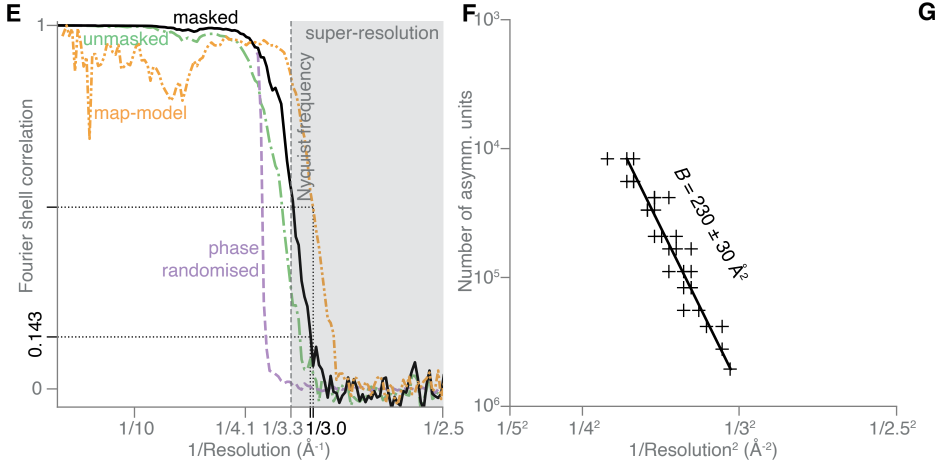

G

Number of 1k × 1k micrographs	402
Number of picked particles	16,458
Number of final particles	8,565
Nominal maginfication	250,000×
Pixel size (phys. & super-res.)	1.657 Å, 0.828 Å
Defocus range	0.2 - 1.0 µm
Average defocus	0.6 µm
Exposure time	3.64 s
Number of raw frames	16,384
Fluence per raw frame	1/149 e⁻/pix/fr
Total electron fluence	40.3 e⁻/Å^2
Frames after grouping	64
Fluence per grouped frame	0.63 e⁻/Å^2/fr
Resolution (FSC = 0.143)	3.0 Å
Efficiency of OD	0.9
Rosenthal *B*-factor	230 Å^2
Symmetry imposed	I
PDB code	8PVJ
EMDB code	17968

Fig. S12. Structure determination for AaLS A representative micrograph (**A**), and three selected reference free 2D class averages (**B**) are shown. Scale bars are all 100 Å. The final reconstructed map is shown in **C**, and a Mollewiede equal area plot of the particle orientation distribution on the sphere is shown in **D**. Fourier shell correlation (FSC) plots (**E**) for the two independently refined, unmasked, dose-weighted, half-maps (green dash-dotted curve), phase-randomised dose-weighted half maps (purple dashed curve), the final independently refined, masked, dose-weighted, half-maps (black solid curve), and the map-model FSC after model refinement (orange curve) are shown. A Rosenthal plot (1) of the resolution vs. number of particles is shown in **F**, and a table of the data collection and analysis statistics is shown in **G**.

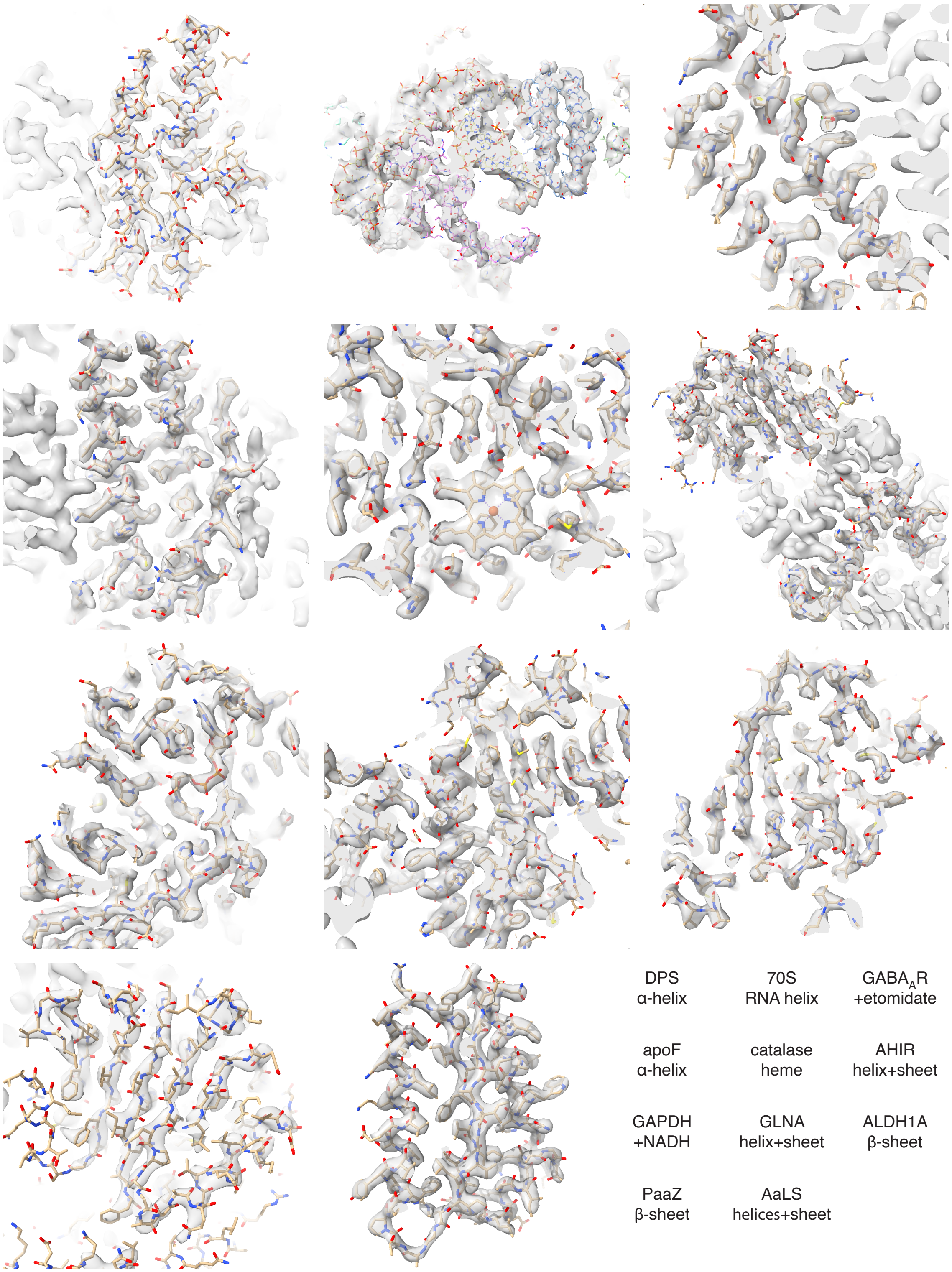

Fig. S13. Structure detail highlights Map-model superpositions showing interesting features in particular structures.

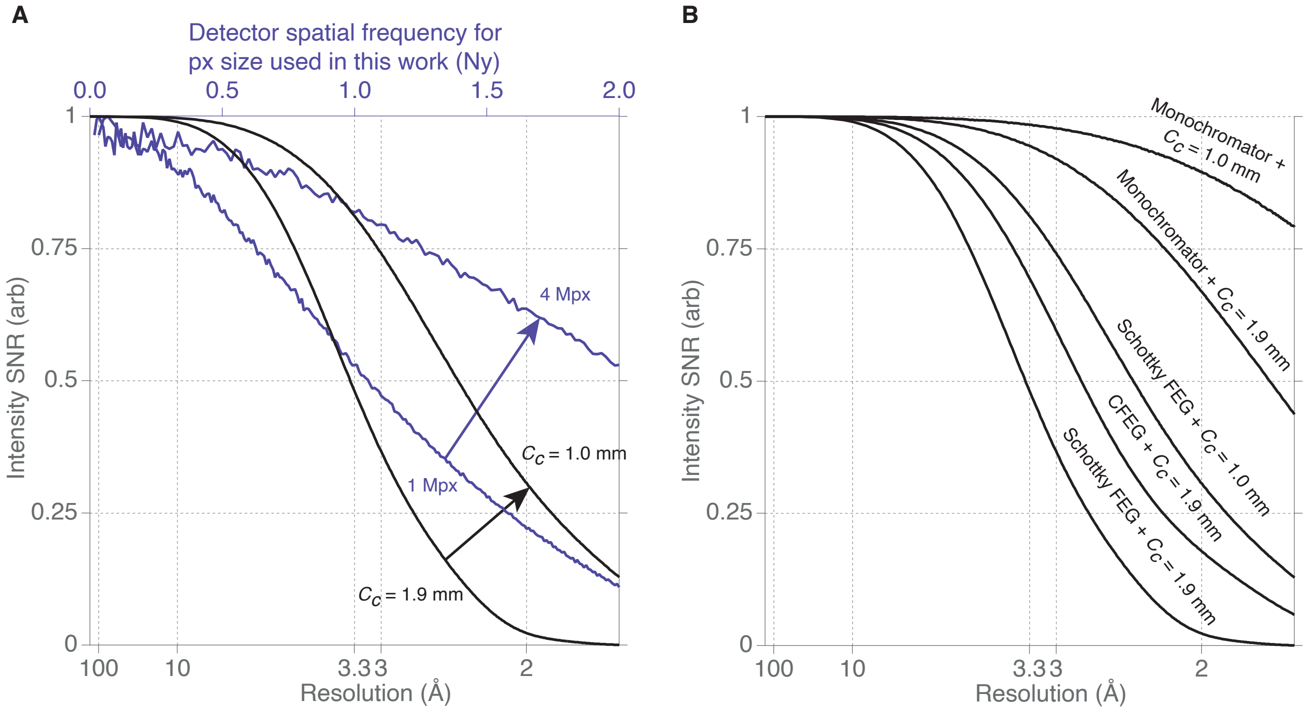

Fig. S14. Potential improvements The potential improvement in the signal intensity to noise ratio (SNR) by reducing the C_c from 1.9 to 1.0 mm and for increasing the number of pixels in the detector from 1k×1k to 2k×2k while keeping the area of the specimen rendered on the detector the same is shown in **A**. The blue lines represent the detector DQE at the current pixel size and with the factor of two increase in magnification enabled by the larger number of pixels on a larger detector. The black curves represent the SNR due to the chromatic envelope function (i.e. amplitude squared) for the measured energy source distribution and the two values of chromatic aberration (1.9 mm current, 1.0 mm future). In **B**, the potential improvement in the temporal coherence envelope relative to the current instrument (Schottky FEG with an energy spread FWHM $= 0.76$ eV + an objective lens with a $C_c = 1.9$ mm) is plotted for several technologies currently available. These include a cold FEG (CFEG) with energy spread FWHM $= 0.38$ eV (2) and a monochromated source with energy spread FWHM = 0.24 (3). Combining a monochromated source with a low C_c lens offers the largest potential gain in signal intensity at 2 Å. Note: each envelope function was calculated using measured energy distributions under realistic imaging conditions used for cryoEM data collection using the method described in section 1.4, as we found that using a Gaussian approximation (as is often done) leads to significant errors in the envelope function that cannot be ignored.

References

1. PB Rosenthal, R Henderson, Optimal determination of particle orientation, absolute hand, and contrast loss in single-particle electron cryomicroscopy. *J Mol Biol* **333**, 721–45 (2003).
2. Y Kohno, et al., Development of a cold field-emission gun for a 200 kV atomic resolution electron microscope. *Microsc. Analysis* **24**, S9–S13 (2010).
3. DC Bell, CJ Russo, G Benner, Sub-Ångstrom low-voltage performance of a monochromated, aberration-corrected transmission electron microscope. *Microsc Microanal* **16**, 386–392 (2010).
